# MRI-Based Radiomics Nomogram: Prediction of Axillary Non-Sentinel Lymph Node Metastasis in Patients With Sentinel Lymph Node-Positive Breast Cancer

**DOI:** 10.3389/fonc.2022.811347

**Published:** 2022-02-28

**Authors:** Ya Qiu, Xiang Zhang, Zhiyuan Wu, Shiji Wu, Zehong Yang, Dongye Wang, Hongbo Le, Jiaji Mao, Guochao Dai, Xuwei Tian, Renbing Zhou, Jiayi Huang, Lanxin Hu, Jun Shen

**Affiliations:** ^1^ Department of Radiology, Sun Yat-Sen Memorial Hospital, Sun Yat-sen University, Guangzhou, China; ^2^ Guangdong Provincial Key Laboratory of Epigenetics and Gene Regulation of Malignant Tumors, Sun Yat-sen Memorial Hospital, Guangzhou, China; ^3^ Department of Radiology, the First People’s Hospital of Kashi Prefecture, Kashi, China; ^4^ School of Public Health, Capital Medical University, Beijing, China; ^5^ Department of Ultrasound, Sun Yat-Sen Memorial Hospital, Sun Yat-Sen University, Guangzhou, China; ^6^ Department of Ultrasound, the First People’s Hospital of Kashi Prefecture, Kashi, China

**Keywords:** multiparametric magnetic resonance imaging, nomograms, sentinel lymph node, lymph node excision, breast neoplasms

## Abstract

**Background:**

Overtreatment of axillary lymph node dissection (ALND) may occur in patients with axillary positive sentinel lymph node (SLN) but negative non-SLN (NSLN). Developing a magnetic resonance imaging (MRI)-based radiomics nomogram to predict axillary NSLN metastasis in patients with SLN-positive breast cancer could effectively decrease the probability of overtreatment and optimize a personalized axillary surgical strategy.

**Methods:**

This retrospective study included 285 patients with positive SLN breast cancer. Fifty five of them had metastatic NSLNs and 230 had non-metastatic NSLNs. MRI-based radiomic features of primary tumors were extracted and MRI morphologic findings of the primary tumor and axillary lymph nodes were assessed. Four models, namely, a radiomics signature, an MRI-clinical nomogram, and two MRI-clinical-radiomics nomograms were established based on MRI morphologic findings, clinicopathologic characteristics, and MRI-based radiomic features to predict the NSLN status. The optimal predictors in each model were selected using the 5-fold cross-validation (CV) method. Their predictive performances were determined by the receiver operating characteristic (ROC) curves analysis. The area under the curves (AUCs) of different models was compared by the Delong test. Their discrimination capability, calibration curve, and clinical usefulness were also assessed.

**Results:**

The 5-fold CV analysis showed that the AUCs ranged from 0.770 to 0.847 for the radiomics signature, from 0.720 to 0.824 for the MRI-clinical nomogram, from 0.843 to 0.932 for the MRI-clinical-radiomics nomogram. The optimal predictive factors in the radiomics signature, MRI-clinical nomogram, and MRI-clinical-radiomics nomogram were one texture feature of diffusion-weighted imaging (DWI), two clinicopathologic features together with one MRI morphologic finding, and the DWI-based texture feature together with the two clinicopathologic features plus the one MRI morphologic finding, respectively. The MRI-clinical-radiomics nomogram with CA 15-3 included achieved the highest AUC compared with the radiomics signature (0.868 *vs*. 0.806, *P <*0.001) and MRI-clinical nomogram (0.868 *vs*. 0.761; *P <*0.001). In addition, the MRI-clinical-radiomics nomogram without CA 15-3 showed a higher performance than that of the radiomics signature (AUC, 0.852 *vs*. 0.806, *P* = 0.016) and the MRI-clinical nomogram (AUC, 0.852 *vs*. 0.761, *P* = 0.007). The MRI-clinical-radiomics nomograms showed good discrimination and good calibration. Decision curve analysis demonstrated that the MRI-clinical-radiomics nomograms were clinically useful.

**Conclusion:**

The MRI-clinical-radiomics nomograms developed in our study showed high predictive performance, which can be used to predict the axillary NSLN status in SLN-positive breast cancer patients before surgery.

## Introduction

Breast cancer is the first high incidence of malignant tumor and the leading cause of death by cancer among female patients (1). Axillary lymph node (ALN) status assessment is of great significance to stage breast cancer and guides the treatment decision-making (2). Nowadays, sentinel lymph node biopsy (SLNB) has substituted for the ALN dissection (ALND) to assess the ALN metastasis in early-stage breast cancer patients (3). Despite a high risk that non-sentinel lymph nodes (NSLNs) metastasis may occur in patients with metastatic sentinel lymph nodes (SLNs) (4, 5), not all patients with a positive SLN would necessarily have a positive NSLN. Indeed, the Z0011 randomized clinical trial showed that only approximately 27.3% of patients with 1 or 2 positive SLNs had NSLN metastasis (6). Other studies showed that 32.1–63% of patients with positive SLNs had NSLNs metastasis, as confirmed by ALND following SLNB (4, 5). These results demonstrate that a considerable number of patients with positive SLN might have negative NSLN; these patients may suffer from overtreatment of ALND (7). Therefore, to avoid unnecessary ALND in a patient with positive SLN but negative NSLN, developing a method to predict the absence or presence of NSLN metastasis is desperately needed.

Previously, several clinicopathologic nomograms (Memorial Sloan Kettering Cancer Center, Mayo, Cambridge, Stanford, and Ljubljana) and scoring systems (Tenon, MD Anderson Cancer Center, and Saidi) have been established to predict the NSLN status (7–14). However, all these models were developed based on pathologic features of the SLN, which could only be obtained from invasive axillary procedures. In addition, except for the Ljubljana nomograms in which preoperative axillary US examination was used as the predictors (7), none of these models have used radiologic features from diagnostic imaging. To date, noninvasive magnetic resonance imaging (MRI) has been recommended as a sufficient tool to comprehensively evaluate ALN status before treatment (15). However, MRI mainly relies on the morphologic criteria to assess the status of the ALN, which showed high specificity but low sensitivity in identifying the ALN metastasis (16). Radiomics could quantify heterogeneity of inter-tumor and intra-tumor by extracting high-throughput data from MR images (17, 18). Previously, MRI-based radiomics of the primary breast cancer has been used to predict the ALN metastasis with an area under the curve (AUC) ranging from 0.81 to 0.92 in training and 0.74 to 0.90 in the validation datasets (19–22), and the SLN burden with a reported AUC of 0.82, 0.81, and 0.81 in the training, validation, and test dataset, respectively (23). However, whether MRI-based radiomics could be applied to predict the NSLN metastasis in breast cancer patients with positive SLNs remains to be determined.

In this study, a large cohort of patients with SLN-positive breast cancer was retrospectively included. Radiomic features of the primary breast tumor on pretreatment multiparametric MRI were extracted, and the MRI-based radiomics signature was constructed to predict the NSLN metastasis. In addition, predictive clinicopathologic features and MRI morphologic findings of breast tumors before treatment were identified to develop an integrative predictive MRI-clinical-radiomics nomogram. The purpose of this study was to develop an MRI-based radiomics model to predict the NSLN metastasis in breast cancer patients with positive SLNs.

## Materials and Methods

### Patients and Study Design

**Figure 1:**
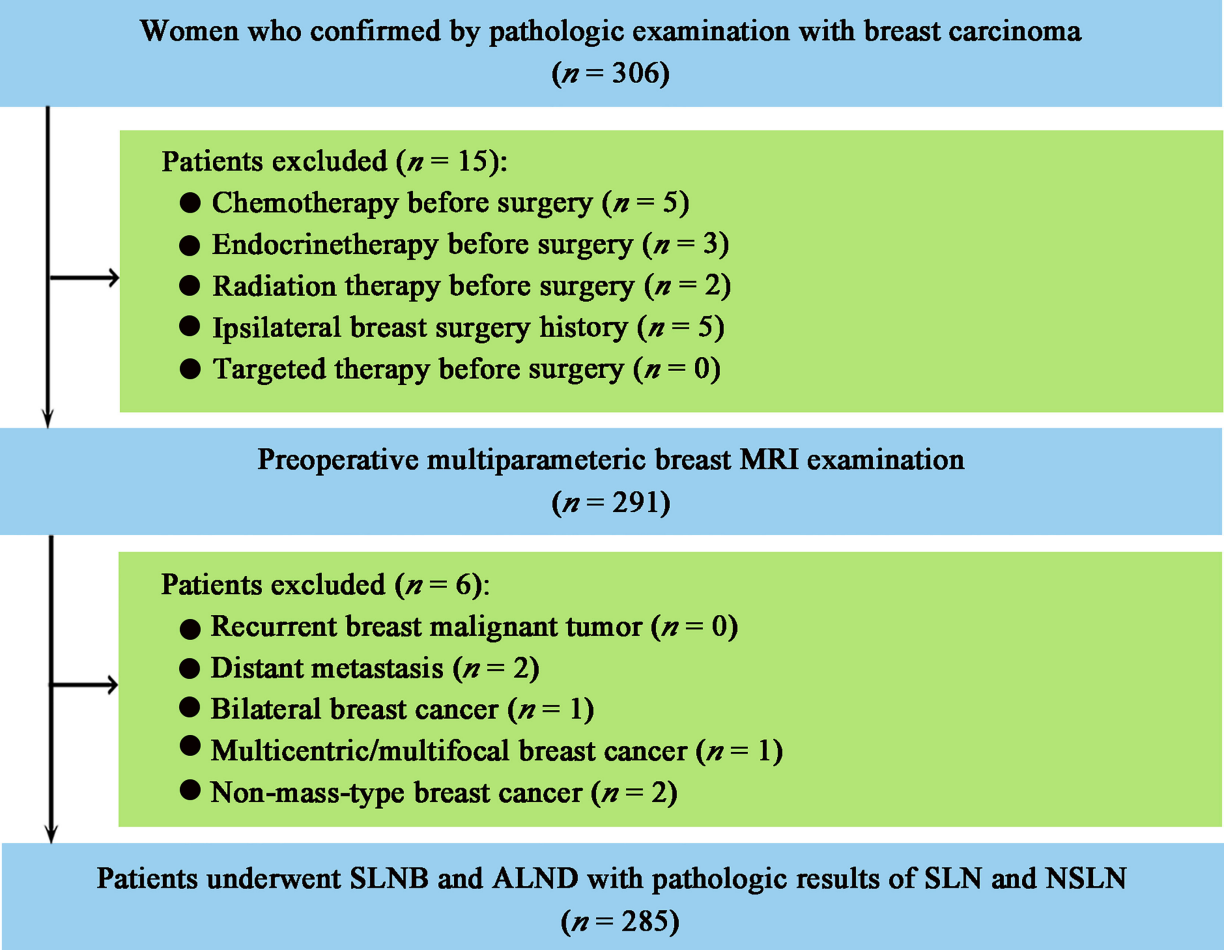
Patient enrollment workflow. MRI, magnetic resonance imaging; SLNB, sentinel lymph node biopsy; ALND, axillary lymph node dissection; SLN, sentinel lymph node; NSLN, non-sentinel lymph node.

This study was approved by the Institutional Review Board of Sun Yat-sen Memorial Hospital, Sun Yat-sen University, and the informed consent was waived because of the nature of the retrospective study. A total of 306 consecutive women with pathologically confirmed primary breast carcinoma were collected from the hospital medical record system between April 2016 and September 2018. The patient enrollment workflow is shown in **Figure 1**. Patients were included if they (i) underwent multiparametric breast MRI examination before breast and axillary surgery; (ii) underwent SLNB and ALND with at least one pathologically positive SLN. The exclusion criteria were as follows: (i) chemotherapy, endocrine therapy, targeted therapy, or radiotherapy before surgery; (ii) recurrent breast malignant tumor; (iii) a history of ipsilateral breast lesion excision; (iv) distant metastasis; and (v) bilateral, multicentric, multifocal, or non-mass-type breast cancer. A total of 285 patients were included. According to the pathologic results of ALND, 285 patients were divided into two groups: the metastatic NSLN group in which at least one NSLN was metastasis (micrometastasis or macrometastasis) pathologically (*n* = 55) and the non-metastatic NSLN group (*n* = 230) in which none of NSLN was metastasis pathologically.

### Clinicopathologic Characteristics

All patients were treated by surgery, namely, breast tumor resection, SLNB, and ALND. SLNB was performed by using the methylene blue technique, as previously described (24). The status of NSLN was identified by ALND and subsequent pathologic examination. The clinicopathologic data, namely, age, family history of breast cancer, palpable breast mass, clinical tumor staging, carcinoembryonic antigen (CEA) level, carbohydrate antigen 15-3 (CA 15-3) level, cytokeratin-19-fragment level, pathologic type of breast cancer, lymphovascular invasion, estrogen receptor (ER) status, progesterone receptor (PR) status, human epidermal growth factor receptor-2 (HER-2) status, Ki-67 status, the number of pathologically proved metastatic SLNs, and the number of pathologically proved metastatic ALNs were collected from the electronic medical record system and pathologic system. Clinical tumor staging was evaluated following the guidelines of the TNM staging system proposed by the American Joint Committee on Cancer (25). In addition, the ALN status determined by preoperative axillary ultrasound (US) examination or US-guided fine-needle aspiration biopsy (FNAB) was collected from the electronic medical record system. The presence of ALN metastasis on US was assessed according to the following abnormal morphologic features: lobulated or eccentric cortex, dislocated and/or absence of fatty hilum, eccentric or concentric thickening ≥2 mm, a cortex-to-hilum ratio ≥1, or a longitudinal axis-to-transverse axis ratio ≤2 (26). During US evaluation, the typical location of the SLN (i.e., axillary tail area) was paid special attention. A biopsy sample was obtained from the most suspicious ALN that showed the above abnormal morphologic characteristics (26).

### Multiparametric MRI Acquisition

MRI was performed on a 1.5 T MR scanner (Magnetom Avanto, Siemens Medical Solutions) with an 8-channel phased-array breast coil (Siemens Medical Solutions). The patients were placed in the prone position with a body parallel to the shoulders, and both breasts were naturally suspended in the coil. The sequences included axial T2-weighted imaging (T2WI), axial T1-weighted imaging (T1WI), axial diffusion-weighted imaging (DWI) with readout segmented echo planar imaging, followed by axial dynamic contrast-enhanced imaging (DCE), axial and coronal delayed contrast-enhanced T1WI (T1 + C). Two dynamic phases of DCE acquisition (40 phases with a temporal resolution of 8 s) were initially performed. And then, all patients underwent intravenous bolus injection of Gd-DTPA-BMA (Omniscan, GE Healthcare; dose = 0.1 mmol/kg body weight; flow rate = 3.5 ml/s) through a high-pressure contrast agent injector (Spectris, Medrad). The T1 + C images were obtained immediately after the DCE imaging was finished. The detailed acquisition parameters are shown in **Table 1**.

**Table 1:** Multiparametric MRI and acquisition parameters.

Sequence	TR/TE (ms)	FOV(mm)	Matrix	Acquisition time (s)	Slice gap (mm)	Fat suppression	Flip angle	Slice thickness (mm)	b value(s/mm^2^)
T2WI	2,500/107	350 × 50	384 × 256	174	1	yes	111°	4	–
T1WI	6.86/2.39	350 × 350	384 × 256	117	1	yes	111°	4	–
DWI	5,400/119	350 × 350	128 × 128	165	1	yes	90°	4	0/800
DCE	4.95/2.28	360 × 360	384 × 224	332	0.8	yes	15°	1.6	–
T1 + C (Axial)	4.85/2.34	360 × 360	320 × 320	65	0.2	yes	5°	1.4	–
T1 + C (Coronal)	6.88/62.39	360 × 360	384 × 384	81	0.4	no	111°	2	–

TR, repetition time; TE, echo time; FOV, field of view; T2WI, T2-weighted imaging; T1WI, T1-weighted imaging; DWI, diffusion-weighted imaging; DCE, dynamic contrast-enhanced imaging; T1+C, contrast-enhanced T1-weighted imaging.

### MRI Morphologic Analysis

Morphologic findings of MRI were assessed by two radiologists (ZY and YQ, with 12 and 7 years of clinical experience in breast MRI diagnosis, respectively) who knew breast cancer diagnosis but were blinded to other clinicopathologic information. All MRI sequences of each patient were available during the morphologic assessment. Any disagreement between the two radiologists was resolved by consultation of another senior radiologist (JS with 20 years of clinical experience in breast MRI diagnosis), and a final diagnosis was made by this senior radiologist. For morphologic analysis, MRI findings, namely, the quadrant of breast cancer, long diameter of breast cancer, presence of ALN metastasis, number of metastatic ALN, and short diameter of the largest ALN, were evaluated. The quadrant of breast cancer and the long diameter of breast cancer were measured on axial or coronal T1 + C image in which the primary tumor showed the largest section. All lymph nodes in the axilla were evaluated on axial and coronal T1 + C images. The ALN metastasis was assessed according to previously morphologic criteria as follows: the disappearance of hilum structure (27), lymphatic hilum displacement, eccentric cortical thickening, short diameter >1 cm, or the ratio of long to a short diameter less than 2 (28). The number of metastatic ALN was recorded. The short diameter of the largest ALN was measured on the axial T1 + C image.

### Radiomic Feature Extraction

**Figure 2:**
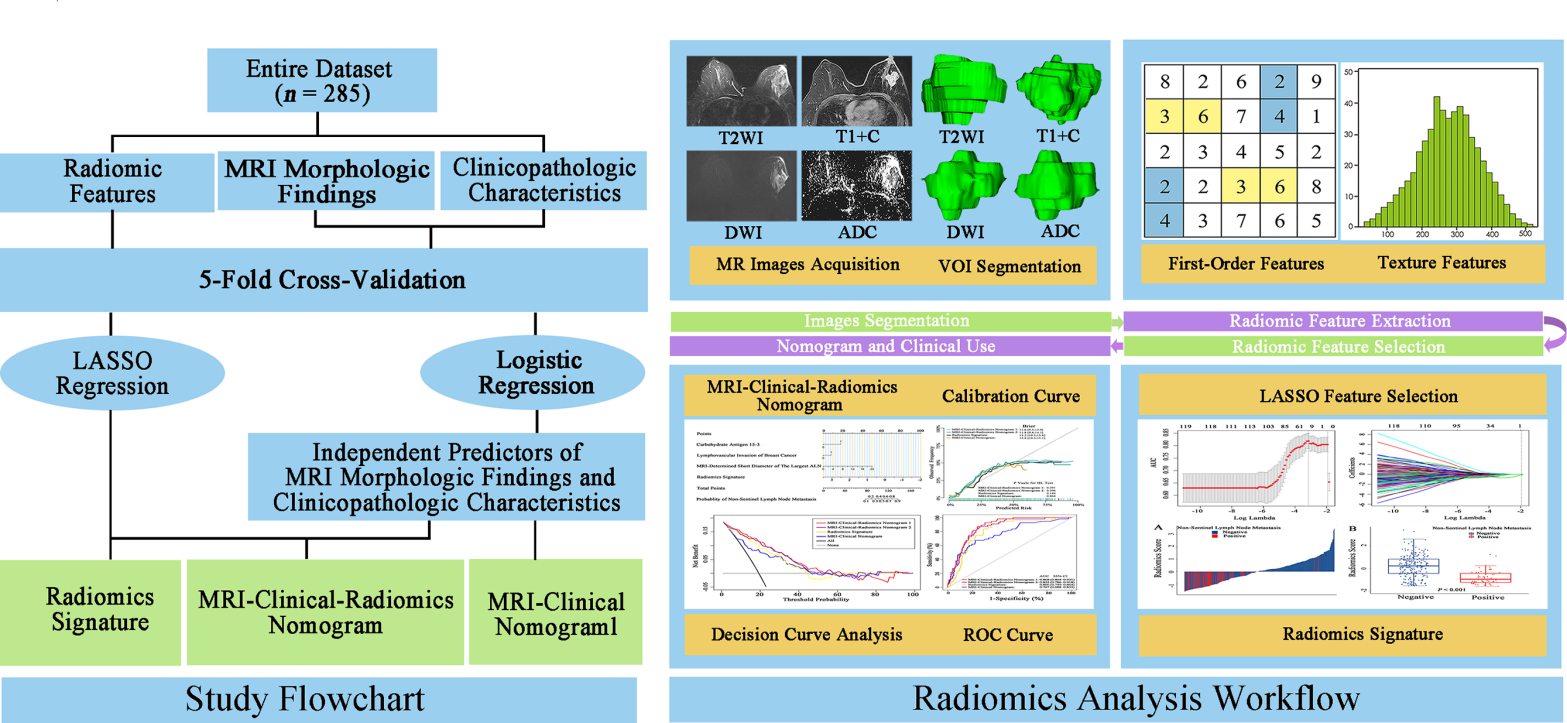
Study flowchart and radiomics analysis workflow. The green rectangular boxes in the study flowchart represent three different non-sentinel lymph node predictive models, namely, radiomics signature, MRI-clinical-radiomics nomogram, and MRI-clinical nomogram. MRI, magnetic resonance imaging; LASSO, shrinkage and selection shrinkage and selection operator; T2WI, T2-weighted imaging; T1 + C, delayed contrast-enhanced T1-weighted imaging; DWI, diffusion-weighted imaging; ADC, apparent diffusion coefficient; VOI, volume of interest.

The flowchart and radiomics analysis workflow are shown in **Figure 2**. First, the primary breast cancer was segmented manually by investigator 1 (XZ, with 10 years of clinical experience in breast MRI diagnosis) to separately create a volume of interest (VOI) on DWI images, apparent diffusion coefficient (ADC) maps, T2WI images, and T1 + C images using the ITK-SNAP (version 3.6.0). Investigator 1 repeated the tumor segmentation in a randomized selecting dataset (*n* = 60) after 2 weeks, and investigator 2 (JH, with 3 years of clinical experience in breast MRI diagnosis) independently performed the segmentation in these 60 patients using the same method as that of investigator 1. Second, radiomic feature extraction was performed using the PyRadiomics toolkit (version 3.0.1) written in Python (version 3.8.3). All the segmented images were interpolated to normalize the spatial resolution in X, Y, and Z directions. For each patient, 1,595 radiomic features were extracted from the initial VOIs and the wavelet filtered, and intensity transformed DWI, ADC, T2WI, and T1 + C images. A total of 6,380 radiomic features were extracted from the primary breast tumors of these four sequences. Details of radiomic features are shown in **Supplementary Table 1**. Third, the intraclass correlation coefficients (ICCs) for the extraction of NSLN metastasis-related radiomic features were assessed by the reproducibility of intra-investigator (first segmentation of investigator 1 vs. second segmentation of investigator 1) and inter-investigator (first segmentation of investigator 1 vs. segmentation of investigator 2), respectively. A good agreement was considered when an ICC was greater than 0.75.

### Development of Predictive Models

MRI morphologic findings, clinicopathologic characteristics, and MRI-based radiomic features were selected to develop three kinds of predictive models, namely, a radiomics signature and two integrative models. For the two integrative predictive models, one was the MRI-clinical nomogram where the independent predictors of MRI morphologic findings and predictive clinicopathologic characteristics were included; the other was the MRI-clinical-radiomics nomogram where the independent predictors of MRI morphologic findings, predictive clinicopathologic characteristics, and radiomics signature were included. To construct integrative predictive models, the Mann–Whitney *U* test was used to compare the MRI morphologic findings and clinicopathologic characteristics between the metastatic NSLN group and the non-metastatic NSLN group. Multivariable logistic regression was then applied to select independent predictors of NSLN metastasis from the MRI morphologic findings and clinicopathologic characteristics. For radiomics analysis, the Mann–Whitney *U* test was performed to select the statistically significant radiomic features between metastatic NSLN group and non-metastatic NSLN group, followed by the least absolute shrinkage and selection operator (LASSO) regression to identify the NSLN metastasis-related radiomic features. The radiomics signature was presented as a radiomics score and constructed by combining the NSLN metastasis-related radiomic features, weighted by the corresponding coefficients of LASSO regression. To determine the optimal independent predictors in each model, a 5-fold cross-validation (CV) analysis was performed by training and testing five separate models to select the most robust predictors (29). For the 5-fold CV analysis, the entire dataset was randomly divided into five subsets, four subsets used for training and another one subset used for testing. This process was repeated five times and five training CV folds and five internal validation CV folds were obtained. The receiver operating characteristic (ROC) curve analysis was used to assess the predictive performance of each model. The area under the curve (AUC) was calculated and compared among different models by the DeLong test (30).

### Performance and Usefulness of Predictive Models

The most robust predictors in the radiomics signature, the MRI-clinical nomogram, and the MRI-clinical-radiomics nomogram selected by the 5-fold CV analysis were used to construct the final predictive models. The performances of the final models of the radiomics signature, MRI-clinical nomogram, and MRI-clinical-radiomics nomogram were determined by the ROC curves analysis in the entire dataset. Their AUCs were compared by the Delong test. The calibration of the final radiomics signature, MRI-clinical nomogram, and MRI-clinical-radiomics nomograms was evaluated using the calibration curves with the Hosmer–Lemeshow test. In addition, the decision curve analysis (DCA) was conducted respectively to assess the clinical use of the final predictive models presenting as the net benefit at different threshold probabilities (31).

### Statistical Analysis

Descriptive statistics were summarized as median (quartile range) for continuous variables or as frequencies with percentages for categorical variables. The continuous variables were compared between different groups by using the *t-*test. The categorical variables were compared between different groups using Pearson’s *χ^2^* or Fisher exact test. The comparison of continuous and categorical variables and ICCs for the feature extraction of intra- and inter-investigator was conducted on SPSS 25. The Mann–Whitney *U* test, LASSO regression, multivariable logistic regression, 5-fold CV, ROC analysis with AUC values calculating, calibration curves, and DCA were performed using the R software (version 4.0.1). *P <*0.05 was considered statistically significant.

## Results

### Clinicopathologic Characteristics and MRI Morphologic Findings

The clinicopathologic characteristics and MRI morphologic findings of 55 patients with metastatic NSLN and 230 patients without metastatic NSLN are summarized in **Table 2**. The time between the breast MRI and surgery ranged from 1 to 12 days, with a median of 5 days. There were significant differences in CA 15-3 status (*P <*0.001), pathologic types of breast cancer (*P* = 0.005), lymphovascular invasion (*P* = 0.001), MRI-determined presence of ALN metastasis (*P* = 0.018), and MRI-determined short diameter of the largest ALN (*P <*0.001) between metastatic and non-metastatic NSLN groups. Most of the patients (272 of 285, 95.4%) had preoperative US results of ALN status. Among these 272 patients, 246 patients had negative results on axillary US examination, and 26 patients had positive results on axillary US examination but negative results US-guided FNAB. Based on the entire dataset of 285 patients, multivariable logistic regression showed that one MR-determined finding (MRI-determined short diameter of the largest ALN), and two clinicopathologic characteristics (CA 15-3 and lymphovascular invasion of breast cancer) were the independent predictors of the NSLN metastasis (**Table 3**). Based on the dataset of 272 patients having preoperative axillary US results, US-reported ALN status was an independent predictor of the NSLN metastasis (**Table 3**). Other MRI morphologic findings and clinicopathologic characteristics were not selected as the independent predictors of the NSLN metastasis (**Supplementary Table 2**).

**Table 2:** Clinicopathologic characteristics and MRI morphologic findings of patients with and without metastatic NSLN.

Characteristic	Non-metastatic NSLN (*n* = 230)	Metastatic NSLN (*n* = 55)	*P*-value
**Age (median, quartile range), years**	49 (44, 58)	50 (45, 59)	0.337^•^
**Family history of breast cancer**			0.578^Δ^
No	227 (98.7)	54 (98.2)	
Yes	3 (1.3)	1 (1.8)	
**Palpable breast mass**			0.028^◊^
No	215 (93.5)	46 (83.6)	
Yes	15 (6.5)	9 (16.4)	
**Clinical tumor staging**			0.100^◊^
T1	117 (50.9)	21 (38.2)	
T2	113 (49.1)	34 (61.8)	
**CEA^#^**			0.738^Δ^
Negative	219 (95.2)	52 (94.5)	
Positive	11 (4.8)	3 (5.5)	
**CA 15-3^#^**			<0.001^◊*^
Negative	218 (94.8)	42 (76.4)	
Positive	12 (5.2)	13 (23.6)	
**CYFR 21-1^#^**			0.063^◊^
Negative	171 (74.3)	34 (61.8)	
Positive	59 (25.7)	21 (38.2)	
**Pathologic type of breast cancer**			0.005^Δ*^
IDC	189 (82.2)	44 (80.0)	
ILC	3 (1.3)	5 (9.1)	
Others^†^	38 (16.5)	6 (10.9)	
**Lymphovascular invasion**			0.001^◊*^
No	181 (78.7)	31 (56.4)	
Yes	49 (21.3)	24 (43.6)	
**ER status**			0.453
Negative	48 (20. 9)	9 (16.4)	
Positive	182 (79.1)	46 (83.6)	
**PR status**			0.546^◊^
Negative	81 (35.2)	17 (30.9)	
Positive	149 (64.8)	38 (69.1)	
**HER-2 status**			0.248^Δ^
Negative	3 (1.3)	2 (3.6)	
Positive	227 (98.7)	53(96.4)	
**Ki-67 status**			0.354^◊^
Negative (<14%)	46 (20)	8 (14.5)	
Positive (≥14%)	184 (80.0)	47 (85.5)	
**MRI-determined quadrant of breast cancer**			0.154^Δ^
Central quadrant	10 (4.3)	1 (1.8)	
Outer-upper quadrant	83 (36.1)	26 (47.8)	
Outer-lower quadrant	42 (18.3)	14 (25.5)	
Upper-inner quadrant	64 (27.8)	8 (14.5)	
Lower-inner quadrant	31 (13.5)	6 (10.9)	
**MRI-determined long diameter of breast cancer (median, quartile range), mm**	19.75 (15.1, 25.7)	22.2 (16.6, 29)	0.074^•^
**MRI-determined presence of ALN metastasis**			0.018^◊^
No	218 (94.8)	46 (83.6)	
Yes	12 (5.2)	9 (16.4)	
**MRI-determined number of metastatic ALN**			0.077^Δ^
<1	218 (94.8)	46 (83.6)	
<2	8 (3.5)	6 (10.9)	
≤3	4 (1.7)	3 (5.5)	
**MRI-determined short diameter of the largest ALN (median, quartile range), mm**	3.60 (2.7,5.3)	5.7 (3.8,8.9)	< 0.001^•*^
**US-reported ALN status** ^‡^			0.041^◊^
Negative	202 (92.2)	44 (83)	
Positive	17 (7.8)	9 (17)	

Numbers in the parentheses were presented as percentages. NSLN, non-sentinel lymph node; CEA, carcinoembryonic antigen; CA 15-3, carbohydrate antigen 15-3, CYFR 21-1, cytokeratin-19-fragment; IDC, invasive ductal carcinoma, ILC, invasive lobular carcinoma; ER, estrogen receptor, PR, progesterone receptor; HER-2, human epidermal growth factor receptor-2; MRI, magnetic resonance imaging; mm, millimeter; ALN, axillary lymph node; US, ultrasound.

^†^Others include intraductal papillary carcinoma, ductal carcinoma in situ, lobular carcinoma in situ, neuroendocrine carcinoma, mucinous carcinoma.

^‡^Data was based on 272 patients who underwent US examination in Sun Yat-sen Memorial Hospital, Sun Yat-sen University.

^#^Laboratory analysis of CEA, CA 15-3, and CYFR 21-1 were performed through blood tests within 1 week before surgery. CEA level ≤5 ng/ml, CA 15-3 level ≤25 U/ml, and CYFR 21-1 level <3.3 ng/ml were set as the normal ranges.

^•^Continuous variables were compared by using the Nonparametric test.

^Δ^Categorical variables were compared by using the Fisher exact test.

^◊^Categorical variables were compared by using Pearson’s χ^2^ test.

*P-value <0.05.

**Table 3:** Multivariate logistic regression analysis of predictors of NSLN metastasis prediction in patients with breast cancer based on entire dataset.

Variables	*β*	Odds ratio (95% CI)^Δ^	*P-*value
MRI-determined short diameter of the largest ALN	0.342	1.408 (1.195–1.658)	<0.001*
US-reported ALN status^‡^	1.829	6.227 (1.871–20.727)	0.003^*^
CA 15-3	2.006	7.436 (2.237–24.719)	0.001*
Lymphovascular invasion of breast cancer	1.612	5.012 (2.213–11.355)	<0.001*

CI, confidence interval; MRI, magnetic resonance imaging; ALN, axillary lymph node; CA 15-3, carbohydrate antigen 15-3; US, ultrasound.

^Δ^Data in parentheses are 95% confidence intervals.

^‡^ Data was based on 272 patients who had preoperative axillary US results.

^*^P-value < 0.05.

### Radiomic Feature Extraction

A total of 6,380 radiomic features were extracted from DWI, ADC, T2WI, and T1 + C images of the primary breast tumors for each patient. The ICCs of these radiomic features ranged from 0.797 to 0.981 and 0.773 to 0.976 for intra- and inter-investigator segmentation, respectively, indicating a good reproducibility for radiomic feature extraction.

### Development of Different Predictive Models

For the radiomics signature, the MRI-clinical nomogram and the MRI-clinical-radiomics nomogram, the selected independent predictors and their AUCs in each training and internal validation CV fold of the 5-fold CV analysis are shown in **Table 4**. The AUCs ranged from 0.774 (95% CI, 0.675–0.873) to 0.847 (95% CI, 0.757–0.937) in the training CV fold and from 0.770 (95% CI, 0.654–0.886) to 0.820 (95% CI, 0.749–0.891) in the internal validation CV fold for the radiomics signature, from 0.758 (95% CI, 0.662–0.854) to 0.824 (95% CI, 0.729–0.919) in the training CV fold and from 0.720 (95% CI, 0.598–0.843) to 0.762 (95% CI, 0.685–0.840) in the internal validation CV fold for the MRI-clinical nomogram, and from 0.850 (95% CI, 0.764–0.936) to 0.932 (95% CI, 0.871–0.993) in the training CV fold and from 0.843 (95% CI, 0.745–0.943) to 0.904 (95% CI, 0.849–0.959) in the validation CV fold for the MRI-clinical-radiomics nomogram. The comparisons of the performances among different predictive models in each training CV fold and internal validation CV fold are shown in **Table 5**. The AUCs of the MRI-clinical-radiomics nomogram were higher than those of the radiomics signature (*P* ≤0.001–0.059) and the MRI-clinical nomogram (*P* = 0.003–0.050). Although Fold 1 model of MRI-clinical-radiomics nomogram appeared to perform the best in training and also validation and in comparison with other models, the most robust variables selected by each CV fold were four features, namely, an MRI morphologic finding (short diameter of the largest ALN), two clinicopathologic features (CA 15-3 and lymphovascular invasion of breast cancer), and a texture feature of DWI (DWI_*original_GLDM_Small_Dependence_High_GrayLevel_Emphasis*), which were considered as the optimal independent predictors and used for final model construction.

**Table 4:** Five-fold cross-validation analysis of different predictive models.

Predictive Model	Fold Sequence	Selected Variable	AUC (95% CI)in training CV fold	AUC (95% CI)in internal validation CV fold
**Radiomics signature**	Fold 1	DWI_*Original GLDM Small Dependence High Gray Level Emphasis* ADC_*Wavelet LLH First order 10 Percentile* ADC_*Wavelet HHH NGTDM Contrast* ADC_*Wavelet HHL GLDM Small Dependence Low Gray Level Emphasis*	0.837 (0.755–0.922)	0.820 (0.749–0.891)
	Fold 2	DWI_*Original GLDM Small Dependence High Gray Level Emphasis*	0.774 (0.675–0.873)	0.794 (0.673–0.915)
	Fold 3	DWI_*Original GLDM Small Dependence High Gray Level Emphasis*	0.806 (0.707–0.906)	0.787 (0.676–0.899)
	Fold 4	DWI_*Original GLDM Small Dependence High Gray Level Emphasis* ADC_*Wavelet LLH First order 10 Percentile* ADC_*Wavelet HHH NGTDM Contrast* ADC_*Wavelet HHL GLDM Small Dependence Low Gray Level Emphasis*	0.847 (0.757–0.937)	0.770 (0.654–0.886)
	Fold 5	DWI_*Original GLDM Small Dependence High Gray Level Emphasis*	0.821 (0.729–0.912)	0.787 (0.676–0.899)
**MRI-clinical nomogram**	Fold 1	CA 15-3 Lymphovascular invasion MRI-determined short diameter of the largest ALN	0.758 (0.662–0.854)	0.762 (0.685–0.840)
	Fold 2	CA 15-3 Lymphovascular invasion MRI-determined short diameter of the largest ALN	0.772 (0.673–0.872)	0.745 (0.734–0.950)
	Fold 3	CA 15-3 Lymphovascular invasion MRI-determined short diameter of the largest ALN	0.779 (0.675–0.883)	0.745 (0.628–0.863)
	Fold 4	CA 15-3 CYFR 21-1 Lymphovascular invasion Pathologic type of breast cancer MRI-determined short diameter of the largest ALN	0.824 (0.729–0.919)	0.720 (0.598–0.843)
	Fold 5	CA 15-3 CYFR 21-1 Lymphovascular invasion Pathologic type of breast cancer MRI-determined short diameter of the largest ALN MRI BI-RADS	0.787 (0.690–0.884)	0.745 (0.628–0.863)
**MRI-clinical-radiomics nomogram**	Fold 1	CA 15-3 Lymphovascular invasion MRI-determined short diameter of the largest ALN DWI_*Original GLDM Small Dependence High Gray Level Emphasis* ADC_*Wavelet LLH First order 10 Percentile* ADC_*Wavelet HHH NGTDM Contrast* ADC_*Wavelet HHL GLDM Small Dependence Low Gray Level Emphasis*	0.906 (0.839–0.973)	0.904 (0.849–0.959)
	Fold 2	CA 15-3 Lymphovascular invasion MRI-determined short diameter of the largest ALN DWI_*Original GLDM Small Dependence High Gray Level Emphasis*	0.850 (0.764–0.936)	0.898 (0.808–0.987)
	Fold 3	CA 15-3 Lymphovascular invasion MRI-determined short diameter of the largest ALN DWI_*Original GLDM Small Dependence High Gray Level Emphasis*	0.875 (0.790–0.959)	0.843 (0.745–0.943)
	Fold 4	CA 15-3 CYFR 21-1 Lymphovascular invasion Pathologic type of breast cancer MRI-determined short diameter of the largest ALN DWI_*Original GLDM Small Dependence High Gray Level Emphasis* ADC_*Wavelet LLH First order 10 Percentile* ADC_*Wavelet HHH NGTDM Contrast* ADC_*Wavelet HHL GLDM Small Dependence Low Gray Level Emphasis*	0.929 (0.864–0.994)	0.886 (0.778–0.974)
	Fold 5	CA 15-3 CYFR 21-1 Lymphovascular invasion Pathologic type of breast cancer MRI-determined short diameter of the largest ALN MRI BI-RADS DWI_*Original GLDM Small Dependence High Gray Level Emphasis*	0.932 (0.871–0.993)	0.843 (0.745–0.943)

AUC, area under the curve; CI, confidence interval; CV, cross-validation; DWI, diffusion-weighted imaging; GLDM, Gray Level Dependence Matrix; ADC, apparent diffusion coefficient; NGTDM, Neighbouring Gray Tone Difference Matrix; MRI, magnetic resonance imaging; ALN, axillary lymph node; CA 15-3, carbohydrate antigen 15-3; CYFR 21-1, Cytokeratin-19-fragment; BI-RADS, Breast imaging-reporting and data system.

**Table 5:** Comparisons of predictive performances of different predictive models in 5-fold cross-validation analysis.

Fold Sequence	*P*-Values for Comparison of AUCs in Training CV Fold		*P*-Values for Comparison of AUCs in Internal Validation CV Fold	
	MRI-Clinical-Radiomics Nomogram vs. MRI-Clinical Nomogram	MRI-Clinical-Radiomics Nomogram vs. Radiomics Signature	MRI-Clinical-Radiomics Nomogram vs. MRI-Clinical Nomogram	MRI-Clinical-Radiomics Nomogram vs. Radiomics Signature
Fold 1	0.017*	0.001*	0.007*	0.001*
Fold 2	0.006*	0.059	0.050	0.006*
Fold 3	0.015*	0.044*	0.042*	0.037*
Fold 4	0.004*	0.007*	0.007*	0.007*
Fold 5	0.003*	0.001*	0.042*	0.037*

MRI, magnetic resonance imaging; AUC, area under the curve; MRI, magnetic resonance imaging.

*P-value < 0.05.

### Performance and Clinical Usefulness of Different Predictive Models

**Figure 3:**
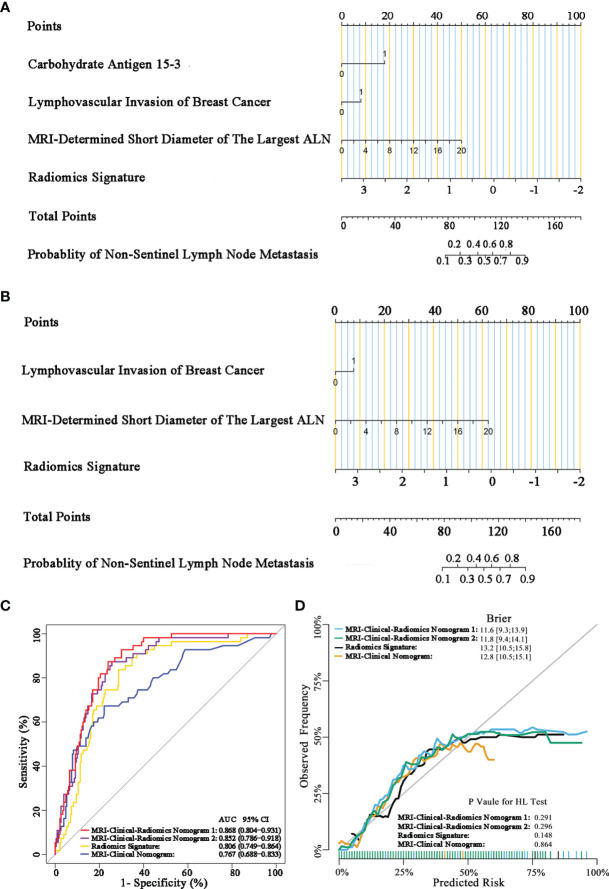
MRI-clinical-radiomics nomograms, receiver operating characteristic (ROC) curves, and calibration curves of predictive models. MRI-clinical-radiomics nomogram **(A)** developed in the entire dataset incorporates one MRI-determined morphologic finding, two clinicopathologic characteristics (lymphovascular invasion of breast cancer plus CA 15-3), and radiomics signature. MRI-clinical-radiomics nomogram **(B)** developed in the entire dataset incorporates one MRI-determined morphologic finding, one clinicopathologic characteristics (lymphovascular invasion of breast cancer alone), and radiomics signature. ROC curves of the radiomics signature, MRI-clinical nomogram, and MRI-clinical-radiomics nomograms with CA 15-3 (MRI-Clinical-Radiomics Nomogram 1) and without CA 15-3 (MRI-Clinical-Radiomics Nomogram 2) in the entire dataset **(C)**. Calibration curves of the radiomics signature, MRI-clinical nomogram, and MRI-clinical-radiomics nomograms in the entire dataset **(D)**. ALN, axillary lymph node; AUC, area under the curve; CI, confidence interval; HL, Hosmer–Lemeshow.

**Figure 4:**
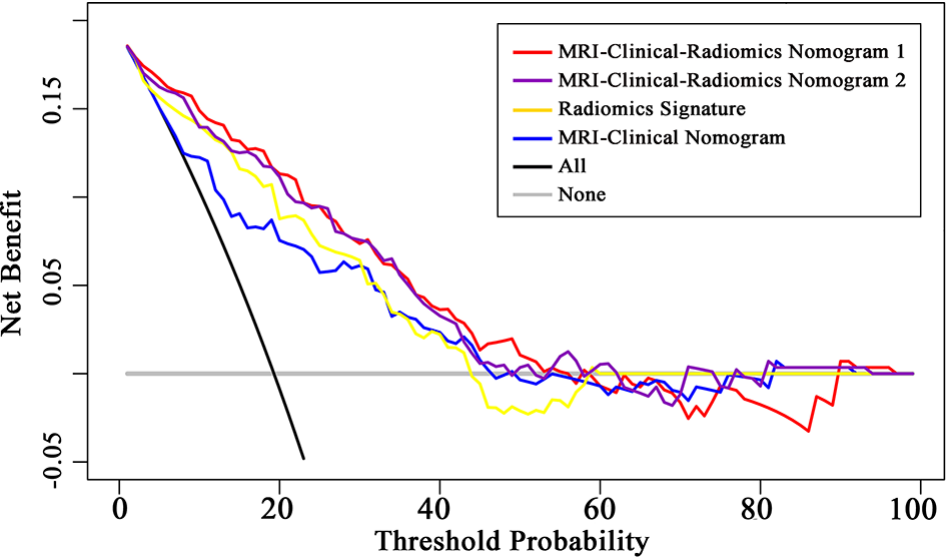
Decision curve analysis (DCA) of the radiomics signature, MRI-clinical nomogram, and MRI-clinical-radiomics nomograms with CA 15-3 (MRI-Clinical-Radiomics Nomogram 1) and without CA 15-3 (MRI-Clinical-Radiomics Nomogram 2). The x-axis and y-axis represent the threshold probability and net benefit, respectively. The gray line and black line represent the hypothesis that all patients and no patient had NSLN metastasis, respectively. The threshold probability is where the expected benefit of treatment is equal to the expected benefit of avoiding treatment. The decision curves in the validation dataset showed that if the threshold probability is between 0.1 and 0.6, using the MRI-clinical-radiomics nomograms to predict non-sentinel lymph node metastasis add more benefit than treating all or treating no patients.

**Figure 5:**
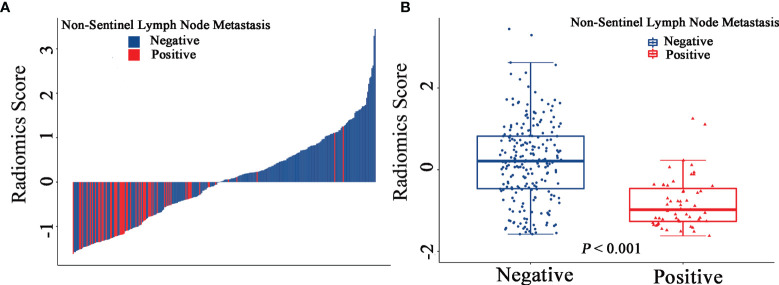
Waterfall plots show the distribution of radiomic feature and non-sentinel lymph node metastasis status for each patient in the entire dataset **(A)**. Boxplots of the radiomic score in the entire dataset **(B)**.

The final model of the MRI-clinical-radiomics nomogram is shown in **Figure 3A**. ROC analysis showed that the final model of the MRI-clinical-radiomics nomogram had an AUC of 0.868, which was significantly higher than that of radiomics signature (0.868 vs. 0.806, *P <*0.001) and MRI-clinical nomogram (0.868 vs. 0.761, *P <*0.001) (**Figure 3B**). As the CA 15-3 is not a standard of care for prediction of NSLN metastasis, the MRI-clinical-radiomics nomogram, namely, an MRI morphologic finding (short diameter of the largest ALN), a clinicopathologic features (lymphovascular invasion of breast cancer), and a texture feature of DWI (DWI_*original_GLDM_Small_Dependence_High_GrayLevel_Emphasis*) but without CA 15-3 were also constructed. This MRI-clinical-radiomics nomogram had an AUC of 0.852, which was significantly higher than those of radiomics signature (0.852 vs. 0.806, *P* = 0.016) and MRI-clinical nomogram (0.852 vs. 0.761, *P* = 0.007) in predicting NSLN metastasis in the entire dataset (**Figure 3C**). The calibration curves (**Figure 3D**) indicated an excellent calibration capability of the MRI-clinical-radiomics nomogram with or without CA 15-3, and the Hosmer–Lemeshow test showed a *P*-value of 0.291 and 0.296, respectively, suggesting a favorable calibration in terms of the agreement between the predicted risk and actual probability for NSLN metastasis. The decision curve analysis showed that if the threshold probability is between 0.1 and 0.6, using the MRI-clinical-radiomics nomograms with or without CA 15-3 to predict NSLN metastasis adds more benefit than either treating-all or treating-no patients (**Figure 4**). Additionally, the radiomics score of each patient is shown in **Figure 5A**. The radiomics scores in the non-metastatic NSLN group were higher than those in the metastatic NSLN group (0.210 [−0.471, 0.822] vs. −0.980 [−1.270, −0.401], *P <*0.001). The comparison of radiomics scores between the two groups is shown in **Figure 5B**.

**Figure 6:**
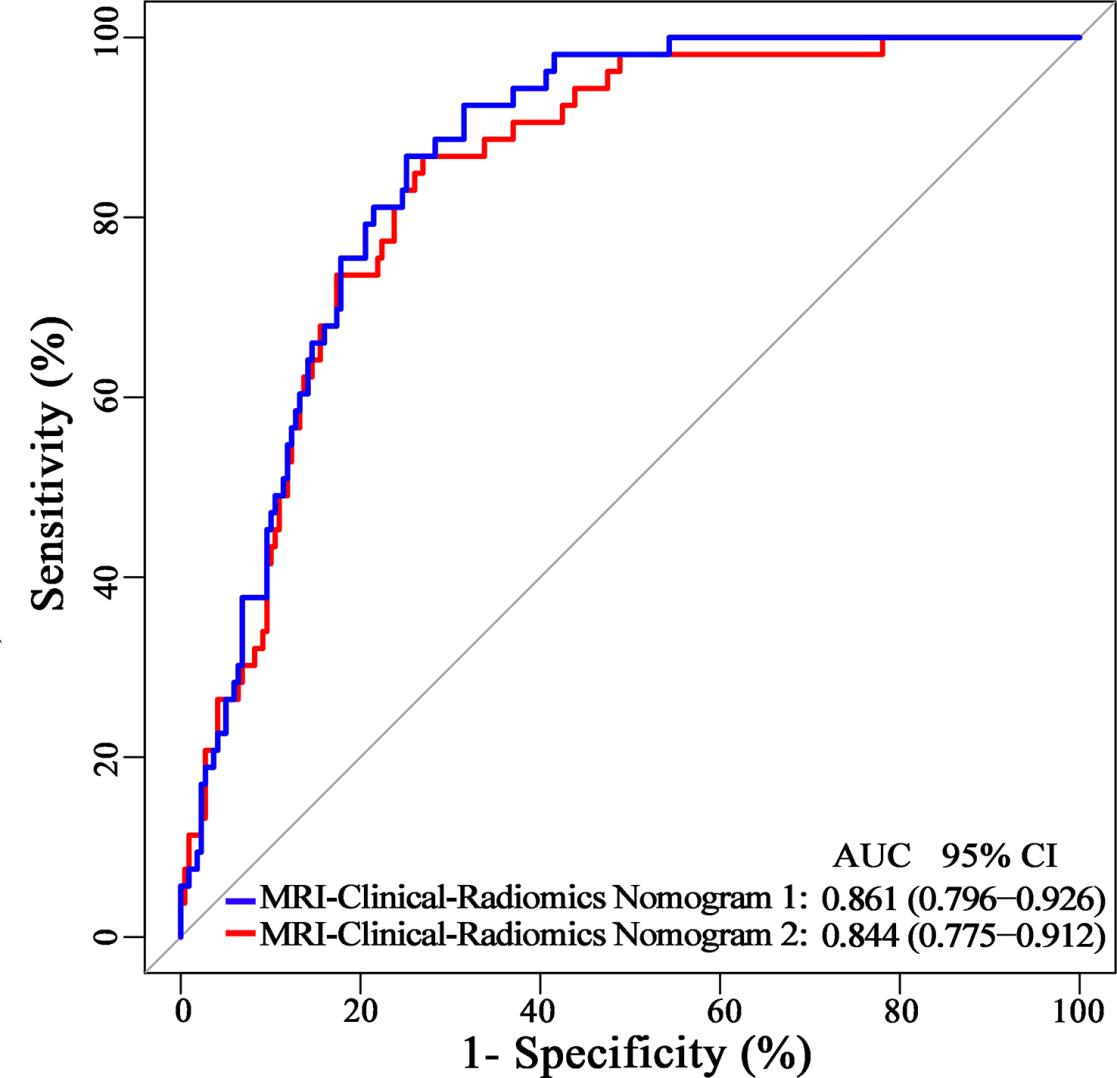
Receiver operating characteristic curves of the MRI-clinical-radiomics nomograms with CA 15-3 (MRI-Clinical-Radiomics Nomogram 1) and without CA 15-3 (MRI-Clinical-Radiomics Nomogram 2) in predicting non-sentinel lymph node metastasis based on 272 patients with negative axillary US examination.

**Figure 7:**
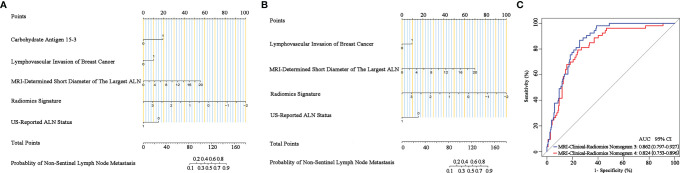
Nomograms, receiver operating characteristic (ROC) curves of the US-reported ALN status-incorporated MRI-clinical-radiomics predictive models with CA 15-3 (MRI-Clinical-Radiomics Nomogram 3) and without CA 15-3 (MRI-Clinical-Radiomics Nomogram 4) in predicting non-sentinel lymph node metastasis based on 272 patients with negative axillary US examination. MRI-clinical-radiomics nomogram 3 **(A)** incorporates one MRI-determined morphologic finding, three clinicopathologic characteristics (lymphovascular invasion of breast cancer, CA 15-3 plus US-reported ALN status), and radiomics signature. MRI-clinical-radiomics nomogram 4 **(B)** incorporates one MRI-determined morphologic finding, two clinicopathologic characteristics (lymphovascular invasion of breast cancer plus US-reported ALN status), and radiomics signature. ROC curves **(C)** of the MRI-Clinical-Radiomics Nomogram 3 and MRI-Clinical-Radiomics Nomogram 4 in predicting non-sentinel lymph node metastasis based on 272 patients with negative axillary US examination. ALN, axillary lymph node; AUC, area under the curve; CI, confidence interval.

Additionally, since axillary US is the most robust axillary assessment tool, the 5-fold cross-validation analysis, where the US-reported ALN status was also included as a variable, was performed in 272 patients with negative axillary US examination (with or without FNAB). The results showed that the US-reported ALN status was not a strong clinical predictor (**Supplementary Table 3**). Based on these 272 patients, the MRI-clinical-radiomics nomograms with CA 15-3 and without CA 15-3 showed an AUC of 0.861 and 0.844 in predicting NSLN metastasis, respectively (**Figure 6**). After the US-reported ALN status was added, the MRI-clinical-radiomics nomograms with CA 15-3 and without CA 15-3 had an AUC of 0.862 and 0.824 in predicting NSLN metastasis in this subcohort (**Figure 7**).

## Discussion

In this study, we developed two MRI-clinical-radiomics nomograms that incorporate one MR-determined finding (short diameter of the largest ALN), one or two clinicopathologic characteristics (i.e. lymphovascular invasion of breast cancer or CA 15-3 plus lymphovascular invasion of breast cancer), and the radiomics signature consisting of one DWI radiomic feature based on the entire dataset of 285 patients. These two MRI-clinical-radiomics nomograms demonstrated robust and high predictive performance (AUC = 0.868 and 0.852), which were both better than the radiomics signature alone and MRI-clinical nomogram. The developed MRI-clinical-radiomics nomograms can serve as novel and easy-to-popularized tools to predict axillary NSLN metastasis in breast cancer patients with positive SLNs.

Invasive ALND is associated with potential postoperative morbidities such as pain, numbness, lymphedema, restricted arm movements, and high risk of infection (32, 33), which can be omitted for those patients at extremely low risk of NSLN metastasis (2). Previously, various clinicopathologic models, such as Memorial Sloan Kettering Cancer Center, Mayo, Cambridge, Stanford, and Ljubljana nomograms, were constructed to predict the NSLN metastasis with reported AUCs range from 0.74 to 0.84 (8–12). It is noted that these predictive models required the pathologic results both from the primary tumor and from the SLN, i.e., the SLN size, the number of positive SLN, and the proportion of positive SLN to all dissected SLN. This information is available only after the invasive SLNB (8–12). In our study, only the preoperative imaging data, clinical details, and pathologic information of the primary breast tumor obtained from biopsy were applied to develop a predictive model. Comparatively, our predictive model may be preferable in clinical practice as it can predict NSLN status without the trauma of the axilla resulting from the SLNB.

To date, a few MRI-based radiomics nomograms have been established for predicting the presence of ALN metastasis, disease-free survival, neoadjuvant chemotherapy efficacy, and tumor microenvironment status in breast cancer patients (19, 34–36). Previously, a Ljubljana nomogram was constructed using the preoperative axillary US features and clinicopathologic information to predict the likelihood of NSLN metastases, with the reported AUCs ranging from 0.75 to 0.79 (7). MRI-based radiomics nomogram to predict the axillary NSLNs metastasis in breast cancer patients with positive SLNs remains a scarcity of data. Dong et al. reported that breast cancer-specific radiomics features extracted from T2WI and DWI images could improve the performance in predicting SLN metastasis, with an AUC of 0.863 in the training set and 0.805 in the validation set (21). In addition, a T2WI and DWI images-based radiomics predictive model could be utilized for preoperative stratification of the SLN low- and heavy-burden in breast cancer patients, yielding an AUC of 0.82, 0.81, and 0.81 in the training, validation, and test dataset, respectively (23). These studies indicated the potential of T2WI- and DWI-based radiomics in predicting the NSLN metastasis. In our study, radiomic features of multiparametric MRI, namely, T2WI, DWI, ADC, and T1 + C were extracted. The 5-fold CV analysis showed that one radiomic feature from DWI (DWI_*original_GLDM_Small_Dependence_High_GrayLevel_Emphasis*) ranged from 0.774 to 0.847 in the training CV fold and from 0.770 to 0.820 in the internal validation CV cohort. Moreover, DWI_*original_GLDM_Small_Dependence_High_GrayLevel_Emphasis* was a consistently selected variable during the 5-fold CV analysis, suggesting that this radiomic feature from DWI was a robust variable. As such, it was selected as the optimal predictor incorporated into the final predictive models. The final model of the one DWI feature-based radiomics signature had a favorable AUC of 0.806 in the entire cohort. This result suggested that the predictive capacity of radiomics features from DWI may be better than the radiomics features extracted from other sequences for predicting the NSLN metastasis. Moreover, this one feature-based radiomics signature might be more convenient for clinical use since fewer reproducible radiomic features imply better reproducibility (37).

To further improve the predictive performance of radiomics signature, clinicopathologic information and MRI-determined morphologic findings were also assessed and incorporated to build an integrative radiomics-based predictive model in our study. Besides the radiomics signature, one MRI morphologic finding (short diameter of the largest ALN), and two clinicopathologic characteristics, including CA 15-3, lymphovascular invasion of breast cancer, were identified as the independent predictors by multivariable logistic regression for NSLN metastasis. The final model of the MRI-clinical-radiomics nomogram incorporating these predictors showed a higher performance than that of the radiomics signature (AUC, 0.868 vs. 0.806, *P <*0.001) and the MRI-clinical nomogram (0.868 vs. 0.761, *P <*0.001) in the entire dataset. In addition, the MRI-clinical-radiomics nomogram without CA 15-3 incorporated also showed a higher performance than those of the radiomics signature (AUC, 0.852 vs. 0.806, *P* = 0.016) and the MRI-clinical nomogram (AUC, 0.852 vs. 0.761, *P* = 0.007) in the entire dataset. It is seemingly that the MRI-clinical-radiomics nomograms developed in our study may serve as a preferable approach to predicting NSLN status in patients with SLN metastasis but without NSLN metastasis. Notably, the MRI-clinical-radiomics nomograms developed in our study also did not need pathologic features that should be obtained from invasive SLNB.

Our study had several limitations. First, the dataset used in our study was retrospectively collected from one center, and no independent external dataset was available for validation, which may limit the generalizability of the radiomics-based nomogram. Further multicenter studies with a larger sample size are needed to acquire high-level evidence for the clinical application of our predictive nomogram. Second, 272 patients (95.4%) underwent preoperative US scan of ALN. Unfortunately, the results of axillary US examination in the remaining 13 patients were not available in our hospital database. This might result in slightly higher than expected SLN involvement in the entire cohort. The accuracy of NSLN prediction could be affected for the constructed predictive models. Third, the proportion of the patients with metastatic NSLN enrolled in our study was relatively small. In our study, the 5-fold CV analysis was used to select the optimal variables for the development of predictive models, as previously reported (29). Forth, manual segmentation of tumors in our study was time- and labor-consuming, which could be improved by a more automatic segmentation approach with the assistance of artificial intelligence in the future. Fifth, the radiomics signature was built based on the radiomic features extracted from primary tumors but not the ALNs. However, it is ambiguous to identify the target ALN for radiomics feature extraction because it has a great challenge to match the ALNs on pathologic examination with the lymph nodes shown on preoperative axillary MRI. Sixth, non-mass-like, multicentric, and multifocal tumors were excluded, which may limit the generalizability of our results. However, it was a great challenge to delineate the boundary of non-mass-like lesions precisely on MR images. In addition, a potential possibility that a heavy burden of axillary NSLN metastasis in patients with multicentric and multifocal tumors may lead to a bias for the patient selection.

In conclusion, two MRI-clinical-radiomics nomograms were developed in our study. The proposed integrative MRI-clinical-radiomics nomograms was one feature-based radiomics signature with one MRI-determined morphologic finding, and one or two clinicopathologic characteristic incorporated, which showed high performance in predicting the axillary NSLN metastasis in patients with SLN positive breast cancer. These MRI-clinical-radiomics nomograms can serve as novel tools to predict axillary NSLN status, which may help avoid unnecessary invasive procedures on the axilla, i.e., ALND, in breast cancer patients with positive SLN but negative NSLN.

## Data Availability Statement

The original contributions presented in the study are included in the article/**Supplementary Material**. Further inquiries can be directed to the corresponding authors.

## Ethics Statement

The studies involving human participants were reviewed and approved by the Institutional Review Board of Sun Yat-Sen Memorial Hospital, Sun Yat-Sen University, and the informed consent was waived because of the nature of the retrospective study. Written informed consent for participation was not required for this study in accordance with the national legislation and the institutional requirements.

## Author Contributions

All authors conceived and execution of this study or analysis of the study data. YQ, XZ, and JS designed the study. YQ, XZ, ZW, SW, DW, HL, JM, GD, XT, RZ, JH, and LH participated in the collection of the clinical information and data analysis. ZW and ZY did the statistical analysis. XZ and JS provided critical comments and suggestions and revised the manuscript. All authors listed have made a substantial, direct, and intellectual contribution to the work and approved it for publication.

## Funding

This work was supported by the Guangdong Province Universities and Colleges Pearl River Scholar Funded Scheme (2017), the National Natural Science Foundation of China (82102130, 8210071257), the Key Areas Research and Development Program of Guangdong (2019B020235001), the Natural Science Foundation of Guangdong Province (2021A1515010385), the Medical Artificial Intelligence Project of Sun Yat-Sen Memorial Hospital (YXRGZN201905), and the Tianshan Youth Project of Xinjiang Uyghur Autonomous Region (2019Q144).

## Conflict of Interest

The authors declare that the research was conducted in the absence of any commercial or financial relationships that could be construed as a potential conflict of interest.

## Publisher’s Note

All claims expressed in this article are solely those of the authors and do not necessarily represent those of their affiliated organizations, or those of the publisher, the editors and the reviewers. Any product that may be evaluated in this article, or claim that may be made by its manufacturer, is not guaranteed or endorsed by the publisher.

## References

[1] Sung H Ferlay J Siegel RL Laversanne M Soerjomataram I Jemal A. Global Cancer Statistics 2020: GLOBOCAN Estimates of Incidence and Mortality Worldwide for 36 Cancers in 185 Countries. CA Cancer J Clin (2021) 71:209–49. doi: 10.3322/caac.21660 33538338

[2] Chang JM Leung JWT Moy L Ha SM Moon WK. Axillary Nodal Evaluation in Breast Cancer: State of the Art. Radiology (2020) 295:500–15. doi: 10.1148/radiol.2020192534 32315268

[3] Lyman GH Somerfield MR Bosserman LD Perkins CL Weaver DL Giuliano AE. Sentinel Lymph Node Biopsy for Patients With Early-Stage Breast Cancer: American Society of Clinical Oncology Clinical Practice Guideline Update. J Clin Oncol (2017) 35:561–4. doi: 10.1200/JCO.2016.71.0947 27937089

[4] Turner RR Chu KU Qi K Botnick LE Hansen NM Glass EC. Pathologic Features Associated With Nonsentinel Lymph Node Metastases in Patients With Metastatic Breast Carcinoma in a Sentinel Lymph Node. Cancer (2000) 89:574–81. doi: 10.1002/1097-0142(20000801) 10931456

[5] Maimaitiaili A Wu D Liu Z Liu H Muyiduli X Fan Z. Analysis of Factors Related to non-Sentinel Lymph Node Metastasis in 296 Sentinel Lymph Node-Positive Chinese Breast Cancer Patients. Cancer Biol Med (2018) 15:282–9. doi: 10.20892/j.issn.2095-3941.2018.0023 PMC6121045 30197795

[6] Giuliano AE Ballman KV McCall L Beitsch PD Brennan MB Kelemen PR. Effect of Axillary Dissection vs No Axillary Dissection on 10-Year Overall Survival Among Women With Invasive Breast Cancer and Sentinel Node Metastasis: The ACOSOG Z0011 (Alliance) Randomized Clinical Trial. JAMA (2017) 318:918–26. doi: 10.1001/jama.2017.11470 PMC5672806 28898379

[7] Perhavec A Perme MP Hocevar M Besić N Zgajnar J. Ljubljana Nomograms for Predicting the Likelihood of Non-Sentinel Lymph Node Metastases in Breast Cancer Patients With a Positive Sentinel Lymph Node. Breast Cancer Res Treat (2010) 119:357–66. doi: 10.1007/s10549-009-0561-4 19787449

[8] Van Zee KJ Manasseh DM Bevilacqua JL Boolbol SK Fey JV Tan LK. A Nomogram for Predicting the Likelihood of Additional Nodal Metastases in Breast Cancer Patients With a Positive Sentinel Node Biopsy. Ann Surg Oncol (2003) 10:1140–51. doi: 10.1245/aso.2003.03.015 14654469

[9] Degnim AC Reynolds C Pantvaidya G Zakaria S Hoskin T Barnes S. Nonsentinel Node Metastasis in Breast Cancer Patients: Assessment of an Existing and a New Predictive Nomogram. Am J Surg (2005) 190:543–50. doi: 10.1016/j.amjsurg.2005.06.008 16164917

[10] Pal A Provenzano E Duffy SW Pinder SE Purushotham AD. A Model for Predicting Non-Sentinel Lymph Node Metastatic Disease When the Sentinel Lymph Node is Positive. Br J Surg (2008) 95:302–9. doi: 10.1002/bjs.5943 17876750

[11] Kohrt HE Olshen RA Bermas HR Goodson WH Wood DJ Henry S. Et Al; New Models and Online Calculator for Predicting Non-Sentinel Lymph Node Status in Sentinel Lymph Node Positive Breast Cancer Patients. BMC Cancer (2008) 8:66. doi: 10.1186/1471-2407-8-66 18315887 PMC2311316

[12] Barranger E Coutant C Flahault A Delpech Y Darai E Uzan S. An Axilla Scoring System to Predict Non-Sentinel Lymph Node Status in Breast Cancer Patients With Sentinel Lymph Node Involvement. Breast Cancer Res Treat (2005) 91:113–9. doi: 10.1007/s10549-004-5781-z 15868438

[13] Hwang RF Krishnamurthy S Hunt KK Mirza N Ames FC Feig B. Clinicopathologic Factors Predicting Involvement of Nonsentinel Axillary Nodes in Women With Breast Cancer. Ann Surg Oncol (2003) 10:248–54. doi: 10.1245/aso.2003.05.020 12679309

[14] Saidi RF Dudrick PS Remine SG Mittal VK. Nonsentinel Lymph Node Status After Positive Sentinel Lymph Node Biopsy in Early Breast Cancer. Am Surg (2004) 70:101–5. 15011910

[15] Byon JH Park YV Yoon JH Moon HJ Kim EK Kim MJ. Added Value of MRI for Invasive Breast Cancer Including the Entire Axilla for Evaluation of High-Level or Advanced Axillary Lymph Node Metastasis in the Post-ACOSOG Z0011 Trial Era. Radiology (2021) 300:46–54. doi: 10.1148/radiol.2021202683 33904772

[16] Zhang X Liu Y Luo H Zhang J. PET/CT and MRI for Identifying Axillary Lymph Node Metastases in Breast Cancer Patients: Systematic Review and Meta-Analysis. J Magn Reson Imaging (2020) 52:1840–51. doi: 10.1002/jmri.27246 32567090

[17] Gillies RJ Kinahan PE Hricak H. Radiomics: Images Are More Than Pictures, They Are Data. Radiology (2016) 278:563–77. doi: 10.1148/radiol.2015151169 PMC4734157 26579733

[18] Lambin P Leijenaar RTH Deist TM Peerlings J de Jong EEC van Timmeren J. Radiomics: The Bridge Between Medical Imaging and Personalized Medicine. Nat Rev Clin Oncol (2017) 14:749–62. doi: 10.1038/nrclinonc.2017.141 28975929

[19] Mao N Dai Y Lin F Ma H Duan S Xie H. Radiomics Nomogram of DCE-MRI for the Prediction of Axillary Lymph Node Metastasis in Breast Cancer. Front Oncol (2020) 10:541849. doi: 10.3389/fonc.2020.541849 33381444 PMC7769044

[20] Liu M Mao N Ma H Dong J Zhang K Che K. Pharmacokinetic Parameters and Radiomics Model Based on Dynamic Contrast Enhanced MRI for the Preoperative Prediction of Sentinel Lymph Node Metastasis in Breast Cancer. Cancer Imaging (2020) 20:65. doi: 10.1186/s40644-020-00342-x 32933585 PMC7493182

[21] Dong Y Feng Q Yang W Lu Z Deng C Zhang L. Preoperative Prediction of Sentinel Lymph Node Metastasis in Breast Cancer Based on Radiomics of T2-Weighted Fat-Suppression and Diffusion-Weighted MRI. Eur Radiol (2018) 28:582–91. doi: 10.1007/s00330-017-5005-7 28828635

[22] Santucci D Faiella E Cordelli E Sicilia R de Felice C Zobel BB. 3t MRI-Radiomic Approach to Predict for Lymph Node Status in Breast Cancer Patients. Cancers (Basel) (2021) 13:2228. doi: 10.3390/cancers13092228 34066451 PMC8124168

[23] Zhang X Yang Z Cui W Zheng C Li H Shen J. Preoperative Prediction of Axillary Sentinel Lymph Node Burden With Multiparametric MRI-Based Radiomics Nomogram in Early-Stage Breast Cancer. Eur Radiol (2021) 31(8):5924–39. doi: 10.1007/s00330-020-07674-z 33569620

[24] Varghese P Abdel-Rahman AT Akberali S Mostafa A Gattuso JM Carpenter R. Methylene Blue Dye–a Safe and Effective Alternative for Sentinel Lymph Node Localization. Breast J (2008) 14:61–7. doi: 10.1111/j.1524-4741 18186867

[25] Kalli S Semine A Cohen S Naber SP Makim SS Bahl M. American Joint Committee on Cancer's Staging System for Breast Cancer, Eighth Edition: What the Radiologist Needs to Know. Radiographics (2018) 38:1921–33. doi: 10.1148/rg.2018180056 30265613

[26] Rautiainen S Masarwah A Sudah M Sutela A Pelkonen O Joukainen S. Axillary Lymph Node Biopsy in Newly Diagnosed Invasive Breast Cancer: Comparative Accuracy of Fine-Needle Aspiration Biopsy Versus Core-Needle Biopsy. Radiology (2013) 269:54–60. doi: 10.1148/radiol.13122637 23771915

[27] Mortellaro VE Marshall J Singer L Hochwald SN Chang M Copeland EM. Magnetic Resonance Imaging for Axillary Staging in Patients With Breast Cancer. J Magn Reson Imaging (2009) 30:309–12. doi: 10.1002/jmri.21802 19466713

[28] Javid S Segara D Lotfi P Raza S Golshan M. Can Breast MRI Predict Axillary Lymph Node Metastasis in Women Undergoing Neoadjuvant Chemotherapy. Ann Surg Oncol (2010) 17:1841–6. doi: 10.1245/s10434-010-0934-2 20143266

[29] Tran D Cooke S Illingworth PJ Gardner DK. Deep Learning as a Predictive Tool for Fetal Heart Pregnancy Following Time-Lapse Incubation and Blastocyst Transfer. Hum Reprod (2019) 34:1011–8. doi: 10.1093/humrep/dez064 PMC6554189 31111884

[30] Demler OV Pencina MJ D'Agostino RB Sr. Misuse of DeLong Test to Compare AUCs for Nested Models. Stat Med (2012) 31:2577–87. doi: 10.1002/sim.5328 PMC3684152 22415937

[31] Wu S Zheng J Li Y Yu H Shi S Xie W. A Radiomics Nomogram for the Preoperative Prediction of Lymph Node Metastasis in Bladder Cancer. Clin Cancer Res (2017) 23:6904–11. doi: 10.1158/1078-0432.CCR-17-1510 28874414

[32] Kootstra JJ Hoekstra-Weebers JE Rietman JS de Vries J Baas PC Geertzen JH. A Longitudinal Comparison of Arm Morbidity in Stage I-II Breast Cancer Patients Treated With Sentinel Lymph Node Biopsy, Sentinel Lymph Node Biopsy Followed by Completion Lymph Node Dissection, or Axillary Lymph Node Dissection. Ann Surg Oncol (2010) 17:2384–94. doi: 10.1245/s10434-010-0981-8 PMC2924495 20221902

[33] Caudle AS Cupp JA Kuerer HM. Management of Axillary Disease. Surg Oncol Clin N Am (2014) 23:473–86. doi: 10.1016/j.soc.2014.03.007 24882346

[34] Yu Y Tan Y Xie C Hu Q Ouyang J Chen Y. Development and Validation of a Preoperative Magnetic Resonance Imaging Radiomics-Based Signature to Predict Axillary Lymph Node Metastasis and Disease-Free Survival in Patients With Early-Stage Breast Cancer. JAMA Netw Open (2020) 3:e2028086. doi: 10.1001/jamanetworkopen.2020.28086 33289845 PMC7724560

[35] Chen S Shu Z Li Y Chen B Tang L Mo W. Machine Learning-Based Radiomics Nomogram Using Magnetic Resonance Images for Prediction of Neoadjuvant Chemotherapy Efficacy in Breast Cancer Patients. Front Oncol (2020) 10:1410. doi: 10.3389/fonc.2020.01410 32923392 PMC7456979

[36] Yu Y He Z Ouyang J Tan Y Chen Y Gu Y. Magnetic Resonance Imaging Radiomics Predicts Preoperative Axillary Lymph Node Metastasis to Support Surgical Decisions and is Associated With Tumor Microenvironment in Invasive Breast Cancer: A Machine Learning, Multicenter Study. EBioMedicine (2021) 69:103460. doi: 10.1016/j.ebiom.2021.103460 34233259 PMC8261009

[37] Sosna J. Fewer Reproducible Radiomic Features Mean Better Reproducibility Within the Same Patient. Radiology (2019) 293:592–3. doi: 10.1148/radiol.2019191958 31577173

