# Supplementary material for: MRI-Based Radiomics Nomogram: Prediction of Axillary Non-Sentinel Lymph Node Metastasis in Patients With Sentinel Lymph Node-Positive Breast Cancer
Source: Front Oncol. 2022 Feb 28;12:811347. doi: 10.3389/fonc.2022.811347 (PMC8920306; doi:10.3389/fonc.2022.811347)
Supplement: Supplementary file 1 [file Table_1.docx]

**Supplementary Material**

**Supplementary Table 1.** Details of radiomic features extracted from T2WI, DWI, ADC and T1+C images

| **Image Transformation** |  | |
| --- | --- | --- |
| **Morphological Features**  **Feature Type** | | **Feature Name** |
| Original  Shape based (*n* =14) | Elongation, Flatness, Least Axis Length, Major Axis Length, Maximum 2D Diameter (Column), Maximum 2D Diameter (Row), Maximum 2D Diameter (Slice), Maximum3D Diameter, Mesh Volume, Minor Axis Length, Sphericity, Surface Area, Surface Volume Ratio, Voxel Volume | |
| **Texture Features**  **Feature Type** | | **Feature Name** |
| Original  First Order Features (*n* = 18) | Energy, Entropy, Interquartile Range, Kurtosis, Maximum, 10 Percentile, 90 Percentile, Mean, Median, Minimum, Mean Absolute Deviation (MAD), Range, Robust Mean Absolute Deviation (rMAD), Root Mean Squared (RMS), Skewness, Total Energy (STE), Uniformity, Variance | |
| Original  Gray Level Co-occurrence Matrix (GLCM) (*n* = 24) | Autocorrelation, Joint Average, Cluster Prominence, Cluster Shade, Cluster Tendency, Contrast, Correlation, Difference Average, Difference Entropy, Difference Variance, Joint Energy, Joint Entropy, Informational Measure of Correlation (IMC) 1, Informational Measure of Correlation (IMC) 2, Inverse Difference Moment (IDM), Maximal Correlation Coefficient (MCC), Inverse Difference Moment Normalized (IDMN), Inverse Difference (ID), Inverse Difference Normalized (IDN), Inverse Variance, Maximum Probability, Sum Average, Sum Entropy, Sum of Squares | |
| Original  Gray Level Dependence Matrix (GLDM) (*n* = 14) | Small Dependence Emphasis (SDE), Large Dependence Emphasis (LDE), Gray Level Non-Uniformity (GLN), Dependence Non-Uniformity (DN), Dependence Non-Uniformity Normalized (DNN), Gray Level Variance (GLV), Dependence Variance (DV), Dependence Entropy (DE), Low Gray Level Emphasis (LGLE), High Gray Level Emphasis (HGLE), Small Dependence Low Gray Level Emphasis (SDLGLE), Small Dependence High Gray Level Emphasis (SDHGLE), Large Dependence Low Gray Level Emphasis (LDLGLE), Large Dependence High Gray Level Emphasis (LDHGLE) | |
| Original  Gray Level Run Length Matrix (GLRLM) (*n* = 16) | Short Run Emphasis (SRE), Long Run Emphasis (LRE), Gray Level Non-Uniformity (GLN), Gray Level Non-Uniformity Normalized (GLNN), Gray Level Variance (GLV), Run Length Non-Uniformity (RLN), Run Length Non-Uniformity Normalized (RLNN), Run Percentage (RP), Run Variance (RV)，Run Entropy (RE), Low Gray Level Run Emphasis (LGLRE), High Gray Level Run Emphasis (HGLRE), Short Run Low Gray Level Emphasis (SRLGLE), Short Run High Gray Level Emphasis (SRHGLE), Long Run Low Gray Level Emphasis (LRLGLE), Long Run High Gray Level Emphasis (LRHGLE) | |
| Original  Gray Level Size Zone Matrix (GLSZM) (*n* = 16) | Small Area Emphasis (SAE), Large Area Emphasis (LAE), Gray Level Non-Uniformity (GLN), Gray Level Non-Uniformity Normalized (GLNN), Size-Zone Non-Uniformity (SZN), Size-Zone Non-Uniformity Normalized (SZNN), Zone Percentage (ZP), Gray Level Variance (GLV), Zone Variance (ZV), Zone Entropy (ZE), Low Gray Level Zone Emphasis (LGLZE), High Gray Level Zone Emphasis (HGLZE), Small Area Low Gray Level Emphasis (SALGLE), Small Area High Gray Level Emphasis (SAHGLE), Large Area Low Gray Level Emphasis (LALGLE), Large Area High Gray Level Emphasis (LAHGLE) | |
| Original  Neighbouring Gray Tone Difference Matrix (NGTDM) (*n* = 5) | Busyness, Coarseness, Complexity, Contrast, Strength | |
| Wavelet LLH  First Order Features (*n* = 18) | Energy, Entropy, Interquartile Range, Kurtosis, Maximum, 10 Percentile, 90 Percentile, Mean, Median, Minimum, Mean Absolute Deviation (MAD), Range, Robust Mean Absolute Deviation (rMAD), Root Mean Squared (RMS), Skewness, Total Energy (STE), Uniformity, Variance | |
| Wavelet LLH  Gray Level Co-occurrence Matrix (GLCM) (*n* = 24) | Autocorrelation, Joint Average, Cluster Prominence, Cluster Shade, Cluster Tendency, Contrast, Correlation, Difference Average, Difference Entropy, Difference Variance, Joint Energy, Joint Entropy, Informational Measure of Correlation (IMC) 1, Informational Measure of Correlation (IMC) 2, Inverse Difference Moment (IDM), Maximal Correlation Coefficient (MCC), Inverse Difference Moment Normalized (IDMN), Inverse Difference (ID), Inverse Difference Normalized (IDN), Inverse Variance, Maximum Probability, Sum Average, Sum Entropy, Sum of Squares | |
| Wavelet LLH  Gray Level Dependence Matrix (GLDM) (*n* = 14) | Small Dependence Emphasis (SDE), Large Dependence Emphasis (LDE), Gray Level Non-Uniformity (GLN), Dependence Non-Uniformity (DN), Dependence Non-Uniformity Normalized (DNN), Gray Level Variance (GLV), Dependence Variance (DV), Dependence Entropy (DE), Low Gray Level Emphasis (LGLE), High Gray Level Emphasis (HGLE), Small Dependence Low Gray Level Emphasis (SDLGLE), Small Dependence High Gray Level Emphasis (SDHGLE), Large Dependence Low Gray Level Emphasis (LDLGLE), Large Dependence High Gray Level Emphasis (LDHGLE) | |
| Wavelet LLH  Gray Level Run Length Matrix (GLRLM) (*n* = 16) | Short Run Emphasis (SRE), Long Run Emphasis (LRE), Gray Level Non-Uniformity (GLN), Gray Level Non-Uniformity Normalized (GLNN), Gray Level Variance (GLV), Run Length Non-Uniformity (RLN), Run Length Non-Uniformity Normalized (RLNN), Run Percentage (RP), Run Variance (RV)，Run Entropy (RE), Low Gray Level Run Emphasis (LGLRE), High Gray Level Run Emphasis (HGLRE), Short Run Low Gray Level Emphasis (SRLGLE), Short Run High Gray Level Emphasis (SRHGLE), Long Run Low Gray Level Emphasis (LRLGLE), Long Run High Gray Level Emphasis (LRHGLE) | |
| Wavelet LLH  Gray Level Size Zone Matrix (GLSZM) (*n* = 16) | Small Area Emphasis (SAE), Large Area Emphasis (LAE), Gray Level Non-Uniformity (GLN), Gray Level Non-Uniformity Normalized (GLNN), Size-Zone Non-Uniformity (SZN), Size-Zone Non-Uniformity Normalized (SZNN), Zone Percentage (ZP), Gray Level Variance (GLV), Zone Variance (ZV), Zone Entropy (ZE), Low Gray Level Zone Emphasis (LGLZE), High Gray Level Zone Emphasis (HGLZE), Small Area Low Gray Level Emphasis (SALGLE), Small Area High Gray Level Emphasis (SAHGLE), Large Area Low Gray Level Emphasis (LALGLE), Large Area High Gray Level Emphasis (LAHGLE) | |
| Wavelet LLH  Neighbouring Gray Tone Difference Matrix (NGTDM) (*n* = 5) | Busyness, Coarseness, Complexity, Contrast, Strength | |
| Wavelet LHL  First Order Features (*n* = 18) | Energy, Entropy, Interquartile Range, Kurtosis, Maximum, 10 Percentile, 90 Percentile, Mean, Median, Minimum, Mean Absolute Deviation (MAD), Range, Robust Mean Absolute Deviation (rMAD), Root Mean Squared (RMS), Skewness, Total Energy (STE), Uniformity, Variance | |
| Wavelet LHL  Gray Level Co-occurrence Matrix (GLCM) (*n* = 24) | Autocorrelation, Joint Average, Cluster Prominence, Cluster Shade, Cluster Tendency, Contrast, Correlation, Difference Average, Difference Entropy, Difference Variance, Joint Energy, Joint Entropy, Informational Measure of Correlation (IMC) 1, Informational Measure of Correlation (IMC) 2, Inverse Difference Moment (IDM), Maximal Correlation Coefficient (MCC), Inverse Difference Moment Normalized (IDMN), Inverse Difference (ID), Inverse Difference Normalized (IDN), Inverse Variance, Maximum Probability, Sum Average, Sum Entropy, Sum of Squares | |
| Wavelet LHL  Gray Level Dependence Matrix (GLDM) (*n* = 14) | Small Dependence Emphasis (SDE), Large Dependence Emphasis (LDE), Gray Level Non-Uniformity (GLN), Dependence Non-Uniformity (DN), Dependence Non-Uniformity Normalized (DNN), Gray Level Variance (GLV), Dependence Variance (DV), Dependence Entropy (DE), Low Gray Level Emphasis (LGLE), High Gray Level Emphasis (HGLE), Small Dependence Low Gray Level Emphasis (SDLGLE), Small Dependence High Gray Level Emphasis (SDHGLE), Large Dependence Low Gray Level Emphasis (LDLGLE), Large Dependence High Gray Level Emphasis (LDHGLE) | |
| Wavelet LHL  Gray Level Run Length Matrix (GLRLM) | Short Run Emphasis (SRE), Long Run Emphasis (LRE), Gray Level Non-Uniformity (GLN), Gray Level Non-Uniformity Normalized (GLNN), Gray Level Variance (GLV), Run Length Non-Uniformity (RLN), Run Length Non-Uniformity Normalized (RLNN), Run Percentage (RP), Run Variance (RV)，Run Entropy (RE), Low Gray Level Run Emphasis (LGLRE), High Gray Level Run Emphasis (HGLRE), Short Run Low Gray Level Emphasis (SRLGLE), Short Run High Gray Level Emphasis (SRHGLE), Long Run Low Gray Level Emphasis (LRLGLE), Long Run High Gray Level Emphasis (LRHGLE) | |
| Wavelet LHL  Gray Level Size Zone Matrix (GLSZM) (*n* = 16) | Small Area Emphasis (SAE), Large Area Emphasis (LAE), Gray Level Non-Uniformity (GLN), Gray Level Non-Uniformity Normalized (GLNN), Size-Zone Non-Uniformity (SZN), Size-Zone Non-Uniformity Normalized (SZNN), Zone Percentage (ZP), Gray Level Variance (GLV), Zone Variance (ZV), Zone Entropy (ZE), Low Gray Level Zone Emphasis (LGLZE), High Gray Level Zone Emphasis (HGLZE), Small Area Low Gray Level Emphasis (SALGLE), Small Area High Gray Level Emphasis (SAHGLE), Large Area Low Gray Level Emphasis (LALGLE), Large Area High Gray Level Emphasis (LAHGLE) | |
| Wavelet LHL  Neighbouring Gray Tone Difference Matrix (NGTDM) (*n* = 5) | Busyness, Coarseness, Complexity, Contrast, Strength | |
| Wavelet LHH  First Order Features (*n* = 18) | Energy, Entropy, Interquartile Range, Kurtosis, Maximum, 10 Percentile, 90 Percentile, Mean, Median, Minimum, Mean Absolute Deviation (MAD), Range, Robust Mean Absolute Deviation (rMAD), Root Mean Squared (RMS), Skewness, Total Energy (STE), Uniformity, Variance | |
| Wavelet LHH  Gray Level Co-occurrence Matrix (GLCM) (*n* = 24) | Autocorrelation, Joint Average, Cluster Prominence, Cluster Shade, Cluster Tendency, Contrast, Correlation, Difference Average, Difference Entropy, Difference Variance, Joint Energy, Joint Entropy, Informational Measure of Correlation (IMC) 1, Informational Measure of Correlation (IMC) 2, Inverse Difference Moment (IDM), Maximal Correlation Coefficient (MCC), Inverse Difference Moment Normalized (IDMN), Inverse Difference (ID), Inverse Difference Normalized (IDN), Inverse Variance, Maximum Probability, Sum Average, Sum Entropy, Sum of Squares | |
| Wavelet LHH  Gray Level Dependence Matrix (GLDM) (*n* = 14) | Small Dependence Emphasis (SDE), Large Dependence Emphasis (LDE), Gray Level Non-Uniformity (GLN), Dependence Non-Uniformity (DN), Dependence Non-Uniformity Normalized (DNN), Gray Level Variance (GLV), Dependence Variance (DV), Dependence Entropy (DE), Low Gray Level Emphasis (LGLE), High Gray Level Emphasis (HGLE), Small Dependence Low Gray Level Emphasis (SDLGLE), Small Dependence High Gray Level Emphasis (SDHGLE), Large Dependence Low Gray Level Emphasis (LDLGLE), Large Dependence High Gray Level Emphasis (LDHGLE) | |
| Wavelet LHH  Gray Level Run Length Matrix (GLRLM) (*n* = 16) | Short Run Emphasis (SRE), Long Run Emphasis (LRE), Gray Level Non-Uniformity (GLN), Gray Level Non-Uniformity Normalized (GLNN), Gray Level Variance (GLV), Run Length Non-Uniformity (RLN), Run Length Non-Uniformity Normalized (RLNN), Run Percentage (RP), Run Variance (RV)，Run Entropy (RE), Low Gray Level Run Emphasis (LGLRE), High Gray Level Run Emphasis (HGLRE), Short Run Low Gray Level Emphasis (SRLGLE), Short Run High Gray Level Emphasis (SRHGLE), Long Run Low Gray Level Emphasis (LRLGLE), Long Run High Gray Level Emphasis (LRHGLE) | |
| Wavelet LHH  Gray Level Size Zone Matrix (GLSZM) (*n* = 16) | Small Area Emphasis (SAE), Large Area Emphasis (LAE), Gray Level Non-Uniformity (GLN), Gray Level Non-Uniformity Normalized (GLNN), Size-Zone Non-Uniformity (SZN), Size-Zone Non-Uniformity Normalized (SZNN), Zone Percentage (ZP), Gray Level Variance (GLV), Zone Variance (ZV), Zone Entropy (ZE), Low Gray Level Zone Emphasis (LGLZE), High Gray Level Zone Emphasis (HGLZE), Small Area Low Gray Level Emphasis (SALGLE), Small Area High Gray Level Emphasis (SAHGLE), Large Area Low Gray Level Emphasis (LALGLE), Large Area High Gray Level Emphasis (LAHGLE) | |
| Wavelet LHH  Neighbouring Gray Tone Difference Matrix (NGTDM) (*n* = 5) | Busyness, Coarseness, Complexity, Contrast, Strength | |
| Wavelet HLL  First Order Features (*n* = 18) | Energy, Entropy, Interquartile Range, Kurtosis, Maximum, 10 Percentile, 90 Percentile, Mean, Median, Minimum, Mean Absolute Deviation (MAD), Range, Robust Mean Absolute Deviation (rMAD), Root Mean Squared (RMS), Skewness, Total Energy (STE), Uniformity, Variance | |
| Wavelet HLL  Gray Level Co-occurrence Matrix (GLCM) (*n* = 24) | Autocorrelation, Joint Average, Cluster Prominence, Cluster Shade, Cluster Tendency, Contrast, Correlation, Difference Average, Difference Entropy, Difference Variance, Joint Energy, Joint Entropy, Informational Measure of Correlation (IMC) 1, Informational Measure of Correlation (IMC) 2, Inverse Difference Moment (IDM), Maximal Correlation Coefficient (MCC), Inverse Difference Moment Normalized (IDMN), Inverse Difference (ID), Inverse Difference Normalized (IDN), Inverse Variance, Maximum Probability, Sum Average, Sum Entropy, Sum of Squares | |
| Wavelet HLL  Gray Level Dependence Matrix (GLDM) (*n* = 14) | Small Dependence Emphasis (SDE), Large Dependence Emphasis (LDE), Gray Level Non-Uniformity (GLN), Dependence Non-Uniformity (DN), Dependence Non-Uniformity Normalized (DNN), Gray Level Variance (GLV), Dependence Variance (DV), Dependence Entropy (DE), Low Gray Level Emphasis (LGLE), High Gray Level Emphasis (HGLE), Small Dependence Low Gray Level Emphasis (SDLGLE), Small Dependence High Gray Level Emphasis (SDHGLE), Large Dependence Low Gray Level Emphasis (LDLGLE), Large Dependence High Gray Level Emphasis (LDHGLE) | |
| Wavelet-HLL  Gray Level Run Length Matrix (GLRLM) (*n* = 16) | Short Run Emphasis (SRE), Long Run Emphasis (LRE), Gray Level Non-Uniformity (GLN), Gray Level Non-Uniformity Normalized (GLNN), Gray Level Variance (GLV), Run Length Non-Uniformity (RLN), Run Length Non-Uniformity Normalized (RLNN), Run Percentage (RP), Run Variance (RV)，Run Entropy (RE), Low Gray Level Run Emphasis (LGLRE), High Gray Level Run Emphasis (HGLRE), Short Run Low Gray Level Emphasis (SRLGLE), Short Run High Gray Level Emphasis (SRHGLE), Long Run Low Gray Level Emphasis (LRLGLE), Long Run High Gray Level Emphasis (LRHGLE) | |
| Wavelet HLL  Gray Level Size Zone Matrix (GLSZM) (*n* = 16) | Small Area Emphasis (SAE), Large Area Emphasis (LAE), Gray Level Non-Uniformity (GLN), Gray Level Non-Uniformity Normalized (GLNN), Size-Zone Non-Uniformity (SZN), Size-Zone Non-Uniformity Normalized (SZNN), Zone Percentage (ZP), Gray Level Variance (GLV), Zone Variance (ZV), Zone Entropy (ZE), Low Gray Level Zone Emphasis (LGLZE), High Gray Level Zone Emphasis (HGLZE), Small Area Low Gray Level Emphasis (SALGLE), Small Area High Gray Level Emphasis (SAHGLE), Large Area Low Gray Level Emphasis (LALGLE), Large Area High Gray Level Emphasis (LAHGLE) | |
| Wavelet HLL  Neighbouring Gray Tone Difference Matrix (NGTDM) (*n* = 5) | Busyness, Coarseness, Complexity, Contrast, Strength | |
| Wavelet HLH  First Order Features (*n* = 18) | Energy, Entropy, Interquartile Range, Kurtosis, Maximum, 10 Percentile, 90 Percentile, Mean, Median, Minimum, Mean Absolute Deviation (MAD), Range, Robust Mean Absolute Deviation (rMAD), Root Mean Squared (RMS), Skewness, Total Energy (STE), Uniformity, Variance | |
| Wavelet HLH  Gray Level Co-occurrence Matrix (GLCM) (*n* = 24) | Autocorrelation, Joint Average, Cluster Prominence, Cluster Shade, Cluster Tendency, Contrast, Correlation, Difference Average, Difference Entropy, Difference Variance, Joint Energy, Joint Entropy, Informational Measure of Correlation (IMC) 1, Informational Measure of Correlation (IMC) 2, Inverse Difference Moment (IDM), Maximal Correlation Coefficient (MCC), Inverse Difference Moment Normalized (IDMN), Inverse Difference (ID), Inverse Difference Normalized (IDN), Inverse Variance, Maximum Probability, Sum Average, Sum Entropy, Sum of Squares | |
| Wavelet HLH  Gray Level Dependence Matrix (GLDM) (*n* = 14) | Small Dependence Emphasis (SDE), Large Dependence Emphasis (LDE), Gray Level Non-Uniformity (GLN), Dependence Non-Uniformity (DN), Dependence Non-Uniformity Normalized (DNN), Gray Level Variance (GLV), Dependence Variance (DV), Dependence Entropy (DE), Low Gray Level Emphasis (LGLE), High Gray Level Emphasis (HGLE), Small Dependence Low Gray Level Emphasis (SDLGLE), Small Dependence High Gray Level Emphasis (SDHGLE), Large Dependence Low Gray Level Emphasis (LDLGLE), Large Dependence High Gray Level Emphasis (LDHGLE) | |
| Wavelet HLH  Gray Level Run Length Matrix (GLRLM) (*n* = 16) | Short Run Emphasis (SRE), Long Run Emphasis (LRE), Gray Level Non-Uniformity (GLN), Gray Level Non-Uniformity Normalized (GLNN), Gray Level Variance (GLV), Run Length Non-Uniformity (RLN), Run Length Non-Uniformity Normalized (RLNN), Run Percentage (RP), Run Variance (RV)，Run Entropy (RE), Low Gray Level Run Emphasis (LGLRE), High Gray Level Run Emphasis (HGLRE), Short Run Low Gray Level Emphasis (SRLGLE), Short Run High Gray Level Emphasis (SRHGLE), Long Run Low Gray Level Emphasis (LRLGLE), Long Run High Gray Level Emphasis (LRHGLE) | |
| Wavelet HLH  Gray Level Size Zone Matrix (GLSZM) (*n* = 16) | Small Area Emphasis (SAE), Large Area Emphasis (LAE), Gray Level Non-Uniformity (GLN), Gray Level Non-Uniformity Normalized (GLNN), Size-Zone Non-Uniformity (SZN), Size-Zone Non-Uniformity Normalized (SZNN), Zone Percentage (ZP), Gray Level Variance (GLV), Zone Variance (ZV), Zone Entropy (ZE), Low Gray Level Zone Emphasis (LGLZE), High Gray Level Zone Emphasis (HGLZE), Small Area Low Gray Level Emphasis (SALGLE), Small Area High Gray Level Emphasis (SAHGLE), Large Area Low Gray Level Emphasis (LALGLE), Large Area High Gray Level Emphasis (LAHGLE) | |
| Wavelet HLH  Neighbouring Gray Tone Difference Matrix (NGTDM) (*n* = 5) | Busyness, Coarseness, Complexity, Contrast, Strength | |
| Wavelet HHL  First Order Features (*n* = 18) | Energy, Entropy, Interquartile Range, Kurtosis, Maximum, 10 Percentile, 90 Percentile, Mean, Median, Minimum, Mean Absolute Deviation (MAD), Range, Robust Mean Absolute Deviation (rMAD), Root Mean Squared (RMS), Skewness, Total Energy (STE), Uniformity, Variance | |
| Wavelet HHL  Gray Level Co-occurrence Matrix (GLCM) (*n* = 24) | Autocorrelation, Joint Average, Cluster Prominence, Cluster Shade, Cluster Tendency, Contrast, Correlation, Difference Average, Difference Entropy, Difference Variance, Joint Energy, Joint Entropy, Informational Measure of Correlation (IMC) 1, Informational Measure of Correlation (IMC) 2, Inverse Difference Moment (IDM), Maximal Correlation Coefficient (MCC), Inverse Difference Moment Normalized (IDMN), Inverse Difference (ID), Inverse Difference Normalized (IDN), Inverse Variance, Maximum Probability, Sum Average, Sum Entropy, Sum of Squares | |
| Wavelet HHL  Gray Level Dependence Matrix (GLDM) (*n* = 14) | Small Dependence Emphasis (SDE), Large Dependence Emphasis (LDE), Gray Level Non-Uniformity (GLN), Dependence Non-Uniformity (DN), Dependence Non-Uniformity Normalized (DNN), Gray Level Variance (GLV), Dependence Variance (DV), Dependence Entropy (DE), Low Gray Level Emphasis (LGLE), High Gray Level Emphasis (HGLE), Small Dependence Low Gray Level Emphasis (SDLGLE), Small Dependence High Gray Level Emphasis (SDHGLE), Large Dependence Low Gray Level Emphasis (LDLGLE), Large Dependence High Gray Level Emphasis (LDHGLE) | |
| Wavelet HHL  Gray Level Run Length Matrix (GLRLM) (*n* = 16) | Short Run Emphasis (SRE), Long Run Emphasis (LRE), Gray Level Non-Uniformity (GLN), Gray Level Non-Uniformity Normalized (GLNN), Gray Level Variance (GLV), Run Length Non-Uniformity (RLN), Run Length Non-Uniformity Normalized (RLNN), Run Percentage (RP), Run Variance (RV)，Run Entropy (RE), Low Gray Level Run Emphasis (LGLRE), High Gray Level Run Emphasis (HGLRE), Short Run Low Gray Level Emphasis (SRLGLE), Short Run High Gray Level Emphasis (SRHGLE), Long Run Low Gray Level Emphasis (LRLGLE), Long Run High Gray Level Emphasis (LRHGLE) | |
| Wavelet HHL  Gray Level Size Zone Matrix (GLSZM) (*n* = 16) | Small Area Emphasis (SAE), Large Area Emphasis (LAE), Gray Level Non-Uniformity (GLN), Gray Level Non-Uniformity Normalized (GLNN), Size-Zone Non-Uniformity (SZN), Size-Zone Non-Uniformity Normalized (SZNN), Zone Percentage (ZP), Gray Level Variance (GLV), Zone Variance (ZV), Zone Entropy (ZE), Low Gray Level Zone Emphasis (LGLZE), High Gray Level Zone Emphasis (HGLZE), Small Area Low Gray Level Emphasis (SALGLE), Small Area High Gray Level Emphasis (SAHGLE), Large Area Low Gray Level Emphasis (LALGLE), Large Area High Gray Level Emphasis (LAHGLE) | |
| Wavelet HHL  Neighbouring Gray Tone Difference Matrix (NGTDM) (*n* = 5) | Busyness, Coarseness, Complexity, Contrast, Strength | |
| Wavelet HHH  First Order Features (*n* = 18) | Energy, Entropy, Interquartile Range, Kurtosis, Maximum, 10 Percentile, 90 Percentile, Mean, Median, Minimum, Mean Absolute Deviation (MAD), Range, Robust Mean Absolute Deviation (rMAD), Root Mean Squared (RMS), Skewness, Total Energy (STE), Uniformity, Variance | |
| Wavelet HHH  Gray Level Co-occurrence Matrix (GLCM) (*n* = 24) | Autocorrelation, Joint Average, Cluster Prominence, Cluster Shade, Cluster Tendency, Contrast, Correlation, Difference Average, Difference Entropy, Difference Variance, Joint Energy, Joint Entropy, Informational Measure of Correlation (IMC) 1, Informational Measure of Correlation (IMC) 2, Inverse Difference Moment (IDM), Maximal Correlation Coefficient (MCC), Inverse Difference Moment Normalized (IDMN), Inverse Difference (ID), Inverse Difference Normalized (IDN), Inverse Variance, Maximum Probability, Sum Average, Sum Entropy, Sum of Squares | |
| Wavelet HHH  Gray Level Dependence Matrix (GLDM) (*n* = 14) | Small Dependence Emphasis (SDE), Large Dependence Emphasis (LDE), Gray Level Non-Uniformity (GLN), Dependence Non-Uniformity (DN), Dependence Non-Uniformity Normalized (DNN), Gray Level Variance (GLV), Dependence Variance (DV), Dependence Entropy (DE), Low Gray Level Emphasis (LGLE), High Gray Level Emphasis (HGLE), Small Dependence Low Gray Level Emphasis (SDLGLE), Small Dependence High Gray Level Emphasis (SDHGLE), Large Dependence Low Gray Level Emphasis (LDLGLE), Large Dependence High Gray Level Emphasis (LDHGLE) | |
| Wavelet- HHH  Gray Level Run Length Matrix (GLRLM) (*n* = 16) | Short Run Emphasis (SRE), Long Run Emphasis (LRE), Gray Level Non-Uniformity (GLN), Gray Level Non-Uniformity Normalized (GLNN), Gray Level Variance (GLV), Run Length Non-Uniformity (RLN), Run Length Non-Uniformity Normalized (RLNN), Run Percentage (RP), Run Variance (RV)，Run Entropy (RE), Low Gray Level Run Emphasis (LGLRE), High Gray Level Run Emphasis (HGLRE), Short Run Low Gray Level Emphasis (SRLGLE), Short Run High Gray Level Emphasis (SRHGLE), Long Run Low Gray Level Emphasis (LRLGLE), Long Run High Gray Level Emphasis (LRHGLE) | |
| Wavelet HHH  Gray Level Size Zone Matrix (GLSZM) (*n* = 16) | Small Area Emphasis (SAE), Large Area Emphasis (LAE), Gray Level Non-Uniformity (GLN), Gray Level Non-Uniformity Normalized (GLNN), Size-Zone Non-Uniformity (SZN), Size-Zone Non-Uniformity Normalized (SZNN), Zone Percentage (ZP), Gray Level Variance (GLV), Zone Variance (ZV), Zone Entropy (ZE), Low Gray Level Zone Emphasis (LGLZE), High Gray Level Zone Emphasis (HGLZE), Small Area Low Gray Level Emphasis (SALGLE), Small Area High Gray Level Emphasis (SAHGLE), Large Area Low Gray Level Emphasis (LALGLE), Large Area High Gray Level Emphasis (LAHGLE) | |
| Wavelet HHH  Neighbouring Gray Tone Difference Matrix (NGTDM) (*n* = 5) | Busyness, Coarseness, Complexity, Contrast, Strength | |
| Wavelet LLL  First Order Features(*n* = 18) | Energy, Entropy, Interquartile Range, Kurtosis, Maximum, 10 Percentile, 90 Percentile, Mean, Median, Minimum, Mean Absolute Deviation (MAD), Range, Robust Mean Absolute Deviation (rMAD), Root Mean Squared (RMS), Skewness, Total Energy (STE), Uniformity, Variance | |
| Wavelet LLL  Gray Level Co-occurrence Matrix (GLCM) (*n* = 24) | Autocorrelation, Joint Average, Cluster Prominence, Cluster Shade, Cluster Tendency, Contrast, Correlation, Difference Average, Difference Entropy, Difference Variance, Joint Energy, Joint Entropy, Informational Measure of Correlation (IMC) 1, Informational Measure of Correlation (IMC) 2, Inverse Difference Moment (IDM), Maximal Correlation Coefficient (MCC), Inverse Difference Moment Normalized (IDMN), Inverse Difference (ID), Inverse Difference Normalized (IDN), Inverse Variance, Maximum Probability, Sum Average, Sum Entropy, Sum of Squares | |
| Wavelet LLL  Gray Level Dependence Matrix (GLDM) (*n* = 14) | Small Dependence Emphasis (SDE), Large Dependence Emphasis (LDE), Gray Level Non-Uniformity (GLN), Dependence Non-Uniformity (DN), Dependence Non-Uniformity Normalized (DNN), Gray Level Variance (GLV), Dependence Variance (DV), Dependence Entropy (DE), Low Gray Level Emphasis (LGLE), High Gray Level Emphasis (HGLE), Small Dependence Low Gray Level Emphasis (SDLGLE), Small Dependence High Gray Level Emphasis (SDHGLE), Large Dependence Low Gray Level Emphasis (LDLGLE), Large Dependence High Gray Level Emphasis (LDHGLE) | |
| Wavelet LLL  Gray Level Run Length Matrix (GLRLM) (*n* = 16) | Short Run Emphasis (SRE), Long Run Emphasis (LRE), Gray Level Non-Uniformity (GLN), Gray Level Non-Uniformity Normalized (GLNN), Gray Level Variance (GLV), Run Length Non-Uniformity (RLN), Run Length Non-Uniformity Normalized (RLNN), Run Percentage (RP), Run Variance (RV)，Run Entropy (RE), Low Gray Level Run Emphasis (LGLRE), High Gray Level Run Emphasis (HGLRE), Short Run Low Gray Level Emphasis (SRLGLE), Short Run High Gray Level Emphasis (SRHGLE), Long Run Low Gray Level Emphasis (LRLGLE), Long Run High Gray Level Emphasis (LRHGLE) | |
| Wavelet LLL  Gray Level Size Zone Matrix (GLSZM) (*n* = 16) | Small Area Emphasis (SAE), Large Area Emphasis (LAE), Gray Level Non-Uniformity (GLN), Gray Level Non-Uniformity Normalized (GLNN), Size-Zone Non-Uniformity (SZN), Size-Zone Non-Uniformity Normalized (SZNN), Zone Percentage (ZP), Gray Level Variance (GLV), Zone Variance (ZV), Zone Entropy (ZE), Low Gray Level Zone Emphasis (LGLZE), High Gray Level Zone Emphasis (HGLZE), Small Area Low Gray Level Emphasis (SALGLE), Small Area High Gray Level Emphasis (SAHGLE), Large Area Low Gray Level Emphasis (LALGLE), Large Area High Gray Level Emphasis (LAHGLE) | |
| Wavelet LLL  Neighbouring Gray Tone Difference Matrix (NGTDM) (*n* = 5) | Busyness, Coarseness, Complexity, Contrast, Strength | |
| Square  First Order Features(*n* = 18) | Energy, Entropy, Interquartile Range, Kurtosis, Maximum, 10 Percentile, 90 Percentile, Mean, Median, Minimum, Mean Absolute Deviation (MAD), Range, Robust Mean Absolute Deviation (rMAD), Root Mean Squared (RMS), Skewness, Total Energy (STE), Uniformity, Variance | |
| Square  Gray Level Co-occurrence Matrix (GLCM) (*n* = 24) | Autocorrelation, Joint Average, Cluster Prominence, Cluster Shade, Cluster Tendency, Contrast, Correlation, Difference Average, Difference Entropy, Difference Variance, Joint Energy, Joint Entropy, Informational Measure of Correlation (IMC) 1, Informational Measure of Correlation (IMC) 2, Inverse Difference Moment (IDM), Maximal Correlation Coefficient (MCC), Inverse Difference Moment Normalized (IDMN), Inverse Difference (ID), Inverse Difference Normalized (IDN), Inverse Variance, Maximum Probability, Sum Average, Sum Entropy, Sum of Squares | |
| Square  Gray Level Dependence Matrix (GLDM) (*n* = 14) | Small Dependence Emphasis (SDE), Large Dependence Emphasis (LDE), Gray Level Non-Uniformity (GLN), Dependence Non-Uniformity (DN), Dependence Non-Uniformity Normalized (DNN), Gray Level Variance (GLV), Dependence Variance (DV), Dependence Entropy (DE), Low Gray Level Emphasis (LGLE), High Gray Level Emphasis (HGLE), Small Dependence Low Gray Level Emphasis (SDLGLE), Small Dependence High Gray Level Emphasis (SDHGLE), Large Dependence Low Gray Level Emphasis (LDLGLE), Large Dependence High Gray Level Emphasis (LDHGLE) | |
| Square  Gray Level Run Length Matrix (GLRLM) (*n* = 16) | Short Run Emphasis (SRE), Long Run Emphasis (LRE), Gray Level Non-Uniformity (GLN), Gray Level Non-Uniformity Normalized (GLNN), Gray Level Variance (GLV), Run Length Non-Uniformity (RLN), Run Length Non-Uniformity Normalized (RLNN), Run Percentage (RP), Run Variance (RV)，Run Entropy (RE), Low Gray Level Run Emphasis (LGLRE), High Gray Level Run Emphasis (HGLRE), Short Run Low Gray Level Emphasis (SRLGLE), Short Run High Gray Level Emphasis (SRHGLE), Long Run Low Gray Level Emphasis (LRLGLE), Long Run High Gray Level Emphasis (LRHGLE) | |
| Square  Gray Level Size Zone Matrix (GLSZM) (*n* = 16) | Small Area Emphasis (SAE), Large Area Emphasis (LAE), Gray Level Non-Uniformity (GLN), Gray Level Non-Uniformity Normalized (GLNN), Size-Zone Non-Uniformity (SZN), Size-Zone Non-Uniformity Normalized (SZNN), Zone Percentage (ZP), Gray Level Variance (GLV), Zone Variance (ZV), Zone Entropy (ZE), Low Gray Level Zone Emphasis (LGLZE), High Gray Level Zone Emphasis (HGLZE), Small Area Low Gray Level Emphasis (SALGLE), Small Area High Gray Level Emphasis (SAHGLE), Large Area Low Gray Level Emphasis (LALGLE), Large Area High Gray Level Emphasis (LAHGLE) | |
| Square  Neighbouring Gray Tone Difference Matrix (NGTDM) (*n* = 5) | Busyness, Coarseness, Complexity, Contrast, Strength | |
| Squareroot  First Order Features (*n* = 18) | Energy, Entropy, Interquartile Range, Kurtosis, Maximum, 10 Percentile, 90 Percentile, Mean, Median, Minimum, Mean Absolute Deviation (MAD), Range, Robust Mean Absolute Deviation (rMAD), Root Mean Squared (RMS), Skewness, Total Energy (STE), Uniformity, Variance | |
| Squareroot  Gray Level Co-occurrence Matrix (GLCM) (*n* = 24) | Autocorrelation, Joint Average, Cluster Prominence, Cluster Shade, Cluster Tendency, Contrast, Correlation, Difference Average, Difference Entropy, Difference Variance, Joint Energy, Joint Entropy, Informational Measure of Correlation (IMC) 1, Informational Measure of Correlation (IMC) 2, Inverse Difference Moment (IDM), Maximal Correlation Coefficient (MCC), Inverse Difference Moment Normalized (IDMN), Inverse Difference (ID), Inverse Difference Normalized (IDN), Inverse Variance, Maximum Probability, Sum Average, Sum Entropy, Sum of Squares | |
| Squareroot  Gray Level Dependence Matrix (GLDM) (*n* = 14) | Small Dependence Emphasis (SDE), Large Dependence Emphasis (LDE), Gray Level Non-Uniformity (GLN), Dependence Non-Uniformity (DN), Dependence Non-Uniformity Normalized (DNN), Gray Level Variance (GLV), Dependence Variance (DV), Dependence Entropy (DE), Low Gray Level Emphasis (LGLE), High Gray Level Emphasis (HGLE), Small Dependence Low Gray Level Emphasis (SDLGLE), Small Dependence High Gray Level Emphasis (SDHGLE), Large Dependence Low Gray Level Emphasis (LDLGLE), Large Dependence High Gray Level Emphasis (LDHGLE) | |
| Squareroot  Gray Level Run Length Matrix (GLRLM) (*n* = 16) | Short Run Emphasis (SRE), Long Run Emphasis (LRE), Gray Level Non-Uniformity (GLN), Gray Level Non-Uniformity Normalized (GLNN), Gray Level Variance (GLV), Run Length Non-Uniformity (RLN), Run Length Non-Uniformity Normalized (RLNN), Run Percentage (RP), Run Variance (RV)，Run Entropy (RE), Low Gray Level Run Emphasis (LGLRE), High Gray Level Run Emphasis (HGLRE), Short Run Low Gray Level Emphasis (SRLGLE), Short Run High Gray Level Emphasis (SRHGLE), Long Run Low Gray Level Emphasis (LRLGLE), Long Run High Gray Level Emphasis (LRHGLE) | |
| Squareroot  Gray Level Size Zone Matrix (GLSZM) (*n* = 16) | Small Area Emphasis (SAE), Large Area Emphasis (LAE), Gray Level Non-Uniformity (GLN), Gray Level Non-Uniformity Normalized (GLNN), Size-Zone Non-Uniformity (SZN), Size-Zone Non-Uniformity Normalized (SZNN), Zone Percentage (ZP), Gray Level Variance (GLV), Zone Variance (ZV), Zone Entropy (ZE), Low Gray Level Zone Emphasis (LGLZE), High Gray Level Zone Emphasis (HGLZE), Small Area Low Gray Level Emphasis (SALGLE), Small Area High Gray Level Emphasis (SAHGLE), Large Area Low Gray Level Emphasis (LALGLE), Large Area High Gray Level Emphasis (LAHGLE) | |
| Squareroot  Neighbouring Gray Tone Difference Matrix (NGTDM) (*n* = 5) | Busyness, Coarseness, Complexity, Contrast, Strength | |
| Logarithm  First Order Features(*n* = 18) | Energy, Entropy, Interquartile Range, Kurtosis, Maximum, 10 Percentile, 90 Percentile, Mean, Median, Minimum, Mean Absolute Deviation (MAD), Range, Robust Mean Absolute Deviation (rMAD), Root Mean Squared (RMS), Skewness, Total Energy (STE), Uniformity, Variance | |
| Logarithm  Gray Level Co-occurrence Matrix (GLCM) (*n* = 24) | Autocorrelation, Joint Average, Cluster Prominence, Cluster Shade, Cluster Tendency, Contrast, Correlation, Difference Average, Difference Entropy, Difference Variance, Joint Energy, Joint Entropy, Informational Measure of Correlation (IMC) 1, Informational Measure of Correlation (IMC) 2, Inverse Difference Moment (IDM), Maximal Correlation Coefficient (MCC), Inverse Difference Moment Normalized (IDMN), Inverse Difference (ID), Inverse Difference Normalized (IDN), Inverse Variance, Maximum Probability, Sum Average, Sum Entropy, Sum of Squares | |
| Logarithm  Gray Level Dependence Matrix (GLDM) (*n* = 14) | Small Dependence Emphasis (SDE), Large Dependence Emphasis (LDE), Gray Level Non-Uniformity (GLN), Dependence Non-Uniformity (DN), Dependence Non-Uniformity Normalized (DNN), Gray Level Variance (GLV), Dependence Variance (DV), Dependence Entropy (DE), Low Gray Level Emphasis (LGLE), High Gray Level Emphasis (HGLE), Small Dependence Low Gray Level Emphasis (SDLGLE), Small Dependence High Gray Level Emphasis (SDHGLE), Large Dependence Low Gray Level Emphasis (LDLGLE), Large Dependence High Gray Level Emphasis (LDHGLE) | |
| Logarithm  Gray Level Run Length Matrix (GLRLM) (*n* = 16) | Short Run Emphasis (SRE), Long Run Emphasis (LRE), Gray Level Non-Uniformity (GLN), Gray Level Non-Uniformity Normalized (GLNN), Gray Level Variance (GLV), Run Length Non-Uniformity (RLN), Run Length Non-Uniformity Normalized (RLNN), Run Percentage (RP), Run Variance (RV)，Run Entropy (RE), Low Gray Level Run Emphasis (LGLRE), High Gray Level Run Emphasis (HGLRE), Short Run Low Gray Level Emphasis (SRLGLE), Short Run High Gray Level Emphasis (SRHGLE), Long Run Low Gray Level Emphasis (LRLGLE), Long Run High Gray Level Emphasis (LRHGLE) | |
| Logarithm  Gray Level Size Zone Matrix (GLSZM) (*n* = 16) | Small Area Emphasis (SAE), Large Area Emphasis (LAE), Gray Level Non-Uniformity (GLN), Gray Level Non-Uniformity Normalized (GLNN), Size-Zone Non-Uniformity (SZN), Size-Zone Non-Uniformity Normalized (SZNN), Zone Percentage (ZP), Gray Level Variance (GLV), Zone Variance (ZV), Zone Entropy (ZE), Low Gray Level Zone Emphasis (LGLZE), High Gray Level Zone Emphasis (HGLZE), Small Area Low Gray Level Emphasis (SALGLE), Small Area High Gray Level Emphasis (SAHGLE), Large Area Low Gray Level Emphasis (LALGLE), Large Area High Gray Level Emphasis (LAHGLE) | |
| Logarithm  Neighbouring Gray Tone Difference Matrix (NGTDM) (*n* = 5) | Busyness, Coarseness, Complexity, Contrast, Strength | |
| Exponential  First Order Features(*n* = 18) | Energy, Entropy, Interquartile Range, Kurtosis, Maximum, 10 Percentile, 90 Percentile, Mean, Median, Minimum, Mean Absolute Deviation (MAD), Range, Robust Mean Absolute Deviation (rMAD), Root Mean Squared (RMS), Skewness, Total Energy (STE), Uniformity, Variance | |
| Exponential  Gray Level Co-occurrence Matrix (GLCM) (*n* = 24) | Autocorrelation, Joint Average, Cluster Prominence, Cluster Shade, Cluster Tendency, Contrast, Correlation, Difference Average, Difference Entropy, Difference Variance, Joint Energy, Joint Entropy, Informational Measure of Correlation (IMC) 1, Informational Measure of Correlation (IMC) 2, Inverse Difference Moment (IDM), Maximal Correlation Coefficient (MCC), Inverse Difference Moment Normalized (IDMN), Inverse Difference (ID), Inverse Difference Normalized (IDN), Inverse Variance, Maximum Probability, Sum Average, Sum Entropy, Sum of Squares | |
| Exponential  Gray Level Dependence Matrix (GLDM) (*n* = 16) | Small Dependence Emphasis (SDE), Large Dependence Emphasis (LDE), Gray Level Non-Uniformity (GLN), Dependence Non-Uniformity (DN), Dependence Non-Uniformity Normalized (DNN), Gray Level Variance (GLV), Dependence Variance (DV), Dependence Entropy (DE), Low Gray Level Emphasis (LGLE), High Gray Level Emphasis (HGLE), Small Dependence Low Gray Level Emphasis (SDLGLE), Small Dependence High Gray Level Emphasis (SDHGLE), Large Dependence Low Gray Level Emphasis (LDLGLE), Large Dependence High Gray Level Emphasis (LDHGLE) | |
| Exponential Gray Level Run Length Matrix (GLRLM) | Short Run Emphasis (SRE), Long Run Emphasis (LRE), Gray Level Non-Uniformity (GLN), Gray Level Non-Uniformity Normalized (GLNN), Gray Level Variance (GLV), Run Length Non-Uniformity (RLN), Run Length Non-Uniformity Normalized (RLNN), Run Percentage (RP), Run Variance (RV)，Run Entropy (RE), Low Gray Level Run Emphasis (LGLRE), High Gray Level Run Emphasis (HGLRE), Short Run Low Gray Level Emphasis (SRLGLE), Short Run High Gray Level Emphasis (SRHGLE), Long Run Low Gray Level Emphasis (LRLGLE), Long Run High Gray Level Emphasis (LRHGLE) | |
| Exponential  Gray Level Size Zone Matrix (GLSZM) (*n* = 16) | Small Area Emphasis (SAE), Large Area Emphasis (LAE), Gray Level Non-Uniformity (GLN), Gray Level Non-Uniformity Normalized (GLNN), Size-Zone Non-Uniformity (SZN), Size-Zone Non-Uniformity Normalized (SZNN), Zone Percentage (ZP), Gray Level Variance (GLV), Zone Variance (ZV), Zone Entropy (ZE), Low Gray Level Zone Emphasis (LGLZE), High Gray Level Zone Emphasis (HGLZE), Small Area Low Gray Level Emphasis (SALGLE), Small Area High Gray Level Emphasis (SAHGLE), Large Area Low Gray Level Emphasis (LALGLE), Large Area High Gray Level Emphasis (LAHGLE) | |
| Exponential  Neighbouring Gray Tone Difference Matrix (NGTDM) (*n* = 5) | Busyness, Coarseness, Complexity, Contrast, Strength | |
| Gradient  First Order Features (*n* = 18) | Energy, Entropy, Interquartile Range, Kurtosis, Maximum, 10 Percentile, 90 Percentile, Mean, Median, Minimum, Mean Absolute Deviation (MAD), Range, Robust Mean Absolute Deviation (rMAD), Root Mean Squared (RMS), Skewness, Total Energy (STE), Uniformity, Variance | |
| Gradient  Gray Level Co-occurrence Matrix (GLCM) (*n* = 24) | Autocorrelation, Joint Average, Cluster Prominence, Cluster Shade, Cluster Tendency, Contrast, Correlation, Difference Average, Difference Entropy, Difference Variance, Joint Energy, Joint Entropy, Informational Measure of Correlation (IMC) 1, Informational Measure of Correlation (IMC) 2, Inverse Difference Moment (IDM), Maximal Correlation Coefficient (MCC), Inverse Difference Moment Normalized (IDMN), Inverse Difference (ID), Inverse Difference Normalized (IDN), Inverse Variance, Maximum Probability, Sum Average, Sum Entropy, Sum of Squares | |
| Gradient  Gray Level Dependence Matrix (GLDM) (*n* = 14) | Small Dependence Emphasis (SDE), Large Dependence Emphasis (LDE), Gray Level Non-Uniformity (GLN), Dependence Non-Uniformity (DN), Dependence Non-Uniformity Normalized (DNN), Gray Level Variance (GLV), Dependence Variance (DV), Dependence Entropy (DE), Low Gray Level Emphasis (LGLE), High Gray Level Emphasis (HGLE), Small Dependence Low Gray Level Emphasis (SDLGLE), Small Dependence High Gray Level Emphasis (SDHGLE), Large Dependence Low Gray Level Emphasis (LDLGLE), Large Dependence High Gray Level Emphasis (LDHGLE) | |
| Gradient  Gray Level Run Length Matrix (GLRLM) (*n* = 16) | Short Run Emphasis (SRE), Long Run Emphasis (LRE), Gray Level Non-Uniformity (GLN), Gray Level Non-Uniformity Normalized (GLNN), Gray Level Variance (GLV), Run Length Non-Uniformity (RLN), Run Length Non-Uniformity Normalized (RLNN), Run Percentage (RP), Run Variance (RV)，Run Entropy (RE), Low Gray Level Run Emphasis (LGLRE), High Gray Level Run Emphasis (HGLRE), Short Run Low Gray Level Emphasis (SRLGLE), Short Run High Gray Level Emphasis (SRHGLE), Long Run Low Gray Level Emphasis (LRLGLE), Long Run High Gray Level Emphasis (LRHGLE) | |
| Gradient  Gray Level Size Zone Matrix (GLSZM) (*n* = 16) | Small Area Emphasis (SAE), Large Area Emphasis (LAE), Gray Level Non-Uniformity (GLN), Gray Level Non-Uniformity Normalized (GLNN), Size-Zone Non-Uniformity (SZN), Size-Zone Non-Uniformity Normalized (SZNN), Zone Percentage (ZP), Gray Level Variance (GLV), Zone Variance (ZV), Zone Entropy (ZE), Low Gray Level Zone Emphasis (LGLZE), High Gray Level Zone Emphasis (HGLZE), Small Area Low Gray Level Emphasis (SALGLE), Small Area High Gray Level Emphasis (SAHGLE), Large Area Low Gray Level Emphasis (LALGLE), Large Area High Gray Level Emphasis (LAHGLE) | |
| Gradient  Neighbouring Gray Tone Difference Matrix (NGTDM) (*n* = 5) | Busyness, Coarseness, Complexity, Contrast, Strength | |
| lbp-3D-m1  First Order Features(*n* = 18) | Energy, Entropy, Interquartile Range, Kurtosis, Maximum, 10 Percentile, 90 Percentile, Mean, Median, Minimum, Mean Absolute Deviation (MAD), Range, Robust Mean Absolute Deviation (rMAD), Root Mean Squared (RMS), Skewness, Total Energy (STE), Uniformity, Variance | |
| lbp-3D-m1  Gray Level Co-occurrence Matrix (GLCM) (*n* = 24) | Autocorrelation, Joint Average, Cluster Prominence, Cluster Shade, Cluster Tendency, Contrast, Correlation, Difference Average, Difference Entropy, Difference Variance, Joint Energy, Joint Entropy, Informational Measure of Correlation (IMC) 1, Informational Measure of Correlation (IMC) 2, Inverse Difference Moment (IDM), Maximal Correlation Coefficient (MCC), Inverse Difference Moment Normalized (IDMN), Inverse Difference (ID), Inverse Difference Normalized (IDN), Inverse Variance, Maximum Probability, Sum Average, Sum Entropy, Sum of Squares | |
| lbp-3D-m1  Gray Level Dependence Matrix (GLDM) (*n* = 14) | Small Dependence Emphasis (SDE), Large Dependence Emphasis (LDE), Gray Level Non-Uniformity (GLN), Dependence Non-Uniformity (DN), Dependence Non-Uniformity Normalized (DNN), Gray Level Variance (GLV), Dependence Variance (DV), Dependence Entropy (DE), Low Gray Level Emphasis (LGLE), High Gray Level Emphasis (HGLE), Small Dependence Low Gray Level Emphasis (SDLGLE), Small Dependence High Gray Level Emphasis (SDHGLE), Large Dependence Low Gray Level Emphasis (LDLGLE), Large Dependence High Gray Level Emphasis (LDHGLE) | |
| lbp-3D-m1  Gray Level Run Length Matrix (GLRLM) (*n* = 16) | Short Run Emphasis (SRE), Long Run Emphasis (LRE), Gray Level Non-Uniformity (GLN), Gray Level Non-Uniformity Normalized (GLNN), Gray Level Variance (GLV), Run Length Non-Uniformity (RLN), Run Length Non-Uniformity Normalized (RLNN), Run Percentage (RP), Run Variance (RV)，Run Entropy (RE), Low Gray Level Run Emphasis (LGLRE), High Gray Level Run Emphasis (HGLRE), Short Run Low Gray Level Emphasis (SRLGLE), Short Run High Gray Level Emphasis (SRHGLE), Long Run Low Gray Level Emphasis (LRLGLE), Long Run High Gray Level Emphasis (LRHGLE) | |
| lbp-3D-m1  Gray Level Size Zone Matrix (GLSZM) (*n* = 16) | Small Area Emphasis (SAE), Large Area Emphasis (LAE), Gray Level Non-Uniformity (GLN), Gray Level Non-Uniformity Normalized (GLNN), Size-Zone Non-Uniformity (SZN), Size-Zone Non-Uniformity Normalized (SZNN), Zone Percentage (ZP), Gray Level Variance (GLV), Zone Variance (ZV), Zone Entropy (ZE), Low Gray Level Zone Emphasis (LGLZE), High Gray Level Zone Emphasis (HGLZE), Small Area Low Gray Level Emphasis (SALGLE), Small Area High Gray Level Emphasis (SAHGLE), Large Area Low Gray Level Emphasis (LALGLE), Large Area High Gray Level Emphasis (LAHGLE) | |
| lbp-3D-m1  Neighbouring Gray Tone Difference Matrix (NGTDM) (*n* = 5) | Busyness, Coarseness, Complexity, Contrast, Strength | |
| lbp-3D-m2  First Order Features(*n* = 18) | Energy, Entropy, Interquartile Range, Kurtosis, Maximum, 10 Percentile, 90 Percentile, Mean, Median, Minimum, Mean Absolute Deviation (MAD), Range, Robust Mean Absolute Deviation (rMAD), Root Mean Squared (RMS), Skewness, Total Energy (STE), Uniformity, Variance | |
| lbp-3D-m2  Gray Level Co-occurrence Matrix (GLCM) (*n* = 24) | Autocorrelation, Joint Average, Cluster Prominence, Cluster Shade, Cluster Tendency, Contrast, Correlation, Difference Average, Difference Entropy, Difference Variance, Joint Energy, Joint Entropy, Informational Measure of Correlation (IMC) 1, Informational Measure of Correlation (IMC) 2, Inverse Difference Moment (IDM), Maximal Correlation Coefficient (MCC), Inverse Difference Moment Normalized (IDMN), Inverse Difference (ID), Inverse Difference Normalized (IDN), Inverse Variance, Maximum Probability, Sum Average, Sum Entropy, Sum of Squares | |
| lbp-3D-m2  Gray Level Dependence Matrix (GLDM) (*n* = 14) | Small Dependence Emphasis (SDE), Large Dependence Emphasis (LDE), Gray Level Non-Uniformity (GLN), Dependence Non-Uniformity (DN), Dependence Non-Uniformity Normalized (DNN), Gray Level Variance (GLV), Dependence Variance (DV), Dependence Entropy (DE), Low Gray Level Emphasis (LGLE), High Gray Level Emphasis (HGLE), Small Dependence Low Gray Level Emphasis (SDLGLE), Small Dependence High Gray Level Emphasis (SDHGLE), Large Dependence Low Gray Level Emphasis (LDLGLE), Large Dependence High Gray Level Emphasis (LDHGLE) | |
| lbp-3D-m2  Gray Level Run Length Matrix (GLRLM) (*n* = 16) | Short Run Emphasis (SRE), Long Run Emphasis (LRE), Gray Level Non-Uniformity (GLN), Gray Level Non-Uniformity Normalized (GLNN), Gray Level Variance (GLV), Run Length Non-Uniformity (RLN), Run Length Non-Uniformity Normalized (RLNN), Run Percentage (RP), Run Variance (RV)，Run Entropy (RE), Low Gray Level Run Emphasis (LGLRE), High Gray Level Run Emphasis (HGLRE), Short Run Low Gray Level Emphasis (SRLGLE), Short Run High Gray Level Emphasis (SRHGLE), Long Run Low Gray Level Emphasis (LRLGLE), Long Run High Gray Level Emphasis (LRHGLE) | |
| lbp-3D-m2  Gray Level Size Zone Matrix (GLSZM) (*n* = 16) | Small Area Emphasis (SAE), Large Area Emphasis (LAE), Gray Level Non-Uniformity (GLN), Gray Level Non-Uniformity Normalized (GLNN), Size-Zone Non-Uniformity (SZN), Size-Zone Non-Uniformity Normalized (SZNN), Zone Percentage (ZP), Gray Level Variance (GLV), Zone Variance (ZV), Zone Entropy (ZE), Low Gray Level Zone Emphasis (LGLZE), High Gray Level Zone Emphasis (HGLZE), Small Area Low Gray Level Emphasis (SALGLE), Small Area High Gray Level Emphasis (SAHGLE), Large Area Low Gray Level Emphasis (LALGLE), Large Area High Gray Level Emphasis (LAHGLE) | |
| lbp-3D-m2  Neighbouring Gray Tone Difference Matrix (NGTDM) (*n* = 5) | Busyness, Coarseness, Complexity, Contrast, Strength | |
| lbp-3D-k  First Order Features(*n* = 18) | Energy, Entropy, Interquartile Range, Kurtosis, Maximum, 10 Percentile, 90 Percentile, Mean, Median, Minimum, Mean Absolute Deviation (MAD), Range, Robust Mean Absolute Deviation (rMAD), Root Mean Squared (RMS), Skewness, Total Energy (STE), Uniformity, Variance | |
| lbp-3D-K  Gray Level Co-occurrence Matrix (GLCM) (*n* = 24) | Autocorrelation, Joint Average, Cluster Prominence, Cluster Shade, Cluster Tendency, Contrast, Correlation, Difference Average, Difference Entropy, Difference Variance, Joint Energy, Joint Entropy, Informational Measure of Correlation (IMC) 1, Informational Measure of Correlation (IMC) 2, Inverse Difference Moment (IDM), Maximal Correlation Coefficient (MCC), Inverse Difference Moment Normalized (IDMN), Inverse Difference (ID), Inverse Difference Normalized (IDN), Inverse Variance, Maximum Probability, Sum Average, Sum Entropy, Sum of Squares | |
| lbp-3D-k  Gray Level Dependence Matrix (GLDM) (*n* = 14) | Small Dependence Emphasis (SDE), Large Dependence Emphasis (LDE), Gray Level Non-Uniformity (GLN), Dependence Non-Uniformity (DN), Dependence Non-Uniformity Normalized (DNN), Gray Level Variance (GLV), Dependence Variance (DV), Dependence Entropy (DE), Low Gray Level Emphasis (LGLE), High Gray Level Emphasis (HGLE), Small Dependence Low Gray Level Emphasis (SDLGLE), Small Dependence High Gray Level Emphasis (SDHGLE), Large Dependence Low Gray Level Emphasis (LDLGLE), Large Dependence High Gray Level Emphasis (LDHGLE) | |
| lbp-3D-k Gray Level Run Length Matrix (GLRLM) (*n* = 16) | Short Run Emphasis (SRE), Long Run Emphasis (LRE), Gray Level Non-Uniformity (GLN), Gray Level Non-Uniformity Normalized (GLNN), Gray Level Variance (GLV), Run Length Non-Uniformity (RLN), Run Length Non-Uniformity Normalized (RLNN), Run Percentage (RP), Run Variance (RV)，Run Entropy (RE), Low Gray Level Run Emphasis (LGLRE), High Gray Level Run Emphasis (HGLRE), Short Run Low Gray Level Emphasis (SRLGLE), Short Run High Gray Level Emphasis (SRHGLE), Long Run Low Gray Level Emphasis (LRLGLE), Long Run High Gray Level Emphasis (LRHGLE) | |
| lbp-3D-k  Gray Level Size Zone Matrix (GLSZM) (*n* = 16) | Small Area Emphasis (SAE), Large Area Emphasis (LAE), Gray Level Non-Uniformity (GLN), Gray Level Non-Uniformity Normalized (GLNN), Size-Zone Non-Uniformity (SZN), Size-Zone Non-Uniformity Normalized (SZNN), Zone Percentage (ZP), Gray Level Variance (GLV), Zone Variance (ZV), Zone Entropy (ZE), Low Gray Level Zone Emphasis (LGLZE), High Gray Level Zone Emphasis (HGLZE), Small Area Low Gray Level Emphasis (SALGLE), Small Area High Gray Level Emphasis (SAHGLE), Large Area Low Gray Level Emphasis (LALGLE), Large Area High Gray Level Emphasis (LAHGLE) | |
| lbp-3D-k  Neighbouring Gray Tone Difference Matrix (NGTDM) (*n* = 16) | Busyness, Coarseness, Complexity, Contrast, Strength | |

**Supplementary Table 2.** Multivariate Logistic Regression Analysis of Predictors of NSLN Metastasis Prediction in Patients with Breast Cancer

| **Variables** | ***β*** | **Odds ratio (95% CI)^Δ^** | ***P* value** |
| --- | --- | --- | --- |
| **Age** | 0.028 | 1.029 (0.989-1.069) | 0.156 |
| **Family history of breast cancer** | 0.016 | 1.016 (0.067-15.392) | 0.991 |
| **Palpable breast mass** | 0.726 | 2.068 (0.617-6.934) | 0.239 |
| **Clinical tumor staging** | 0.243 | 1.275 (0.441-3.685) | 0.654 |
| **CEA** | -1.726 | 0.178 (0.025-1.288) | 0.087 |
| **CA 15-3** | 2.006 | 7.436 (2.237-24.719) | 0.001* |
| **CYFR 21-1** | 0.605 | 1.832 (0.795-4.220) | 0.155 |
| **Pathologic type of breast cancer** | 0.452 | 1.572 (0.476-5.189) | 0.458 |
| **Lymphovascular invasion of breast cancer** | 1.612 | 5.012 (2.213-11.355) | < 0.001* |
| **ER status** | 0.638 | 1.892 (0.523-6.848) | 0.331 |
| **PR status** | -0.402 | 0.669 (0.235-1.908) | 0.452 |
| **HER-2 status** | -2.504 | 0.082 (0.010-0.657) | 0.019* |
| **Ki-67 status** | -0.058 | 0.944 (0.339-2.627) | 0.912 |
| **MRI-determined long diameter of breast cancer** | 0.010 | 1.011 (0.946-1.080) | 0.765 |
| **MRI-determined presence of ALN metastasis** | 19.940 | 45703738 (0.000-+∞) | 0.999 |
| **MRI-determined number of metastatic ALN** | -23.472 | 0.000 (0.000-0.000) | 0.999 |
| **MRI-determined short diameter of the largest ALN** | 0.342 | 1.408 (1.195-1.658) | < 0.001* |
| **US-reported ALN status**^‡^ | 1.829 | 6.227 (1.871-20.727) | 0.003* |

NSLN, non-sentinel lymph node; CI: confidence interval; CEA, carcinoembryonic antigen; CA 15-3, carbohydrate antigen 15-3, CYFR 21-1, cytokeratin-19-fragment; ER, estrogen receptor; PR, progesterone receptor; HER-2, human epidermal growth factor receptor-2; MRI, [magnetic resonance imaging](http://fmrs.metstr.com/javascript:void(0);); mm, millimeter; ALN, axillary lymph node; US, ultrasound.

^Δ^: Data in parentheses are 95% confidence intervals.

^‡^ : Data was based on 272 patients who had preoperative axillary US results.

^*^*P* value < 0.05.

**Supplementary Table 3.** Five-Fold Cross-Validation Analysis in Different Predictive Models Based on 272 Patients with Negative Axillary Ultrasound Examination

| **Predictive Model** | **Sequence Number** | **Variable** | **Coefficient** | **λ value** | **AUC (95% CI)**  **in training CV fold** | **AUC (95% CI)**  **in internal validation CV fold** |
| --- | --- | --- | --- | --- | --- | --- |
| **Radiomics signature** | 1 | DWI_ Original GLDM Small Dependence High Gray Level Emphasis  ADC_ Wavelet HHH NGTDM Contrast | -0.44089266  0.08594007 | 0.13875410 | 0.807  (0.725-0.888) | 0.832  (0.661-1.000) |
|  | 2 | DWI_ Original GLDM Small Dependence High Gray Level Emphasis | 0.08893196 | 0.13527380 | 0.792  (0.706-0.878) | 0.802  (0.643-0.962) |
|  | 3 | DWI_ Original GLDM Small Dependence High Gray Level Emphasis | -0.17671750 | 0.13816900 | 0.808  (0.728-0.899) | 0.813  (0.624-1.000) |
|  | 4 | DWI_ Original GLDM Small Dependence High Gray Level Emphasis | -0.08747149 | 0.13505450 | 0.793  (0.706-0.880) | 0.832  (0.686-0.977) |
|  | 5 | DWI_ Original GLDM Small Dependence High Gray Level Emphasis | -0.09391285 | 0.14150350 | 0.786  (0.702-0.872) | 0.816  (0.647-0.984) |
| **MRI-Clinical Nomogram** | 1 | CA 15-3  MRI-determined short diameter of the largest ALN | 0.00241578  0.06460718 | 0.11035530 | 0.608  (0.511-0.705) | 0.533  (0.322-0.744) |
|  | 2 | CA 15-3  Lymphovascular invasion  Pathologic type of breast cancer  MRI-determined short diameter of the largest ALN  MRI BI_RADS | 0.40783246  0.39530084  0.43770842  0.10373059  -0.11705715 | 0.13527380 | 0.771  (0.782-0.860) | 0.791  (0.628-0.953) |
|  | 3 | CA 15-3  MRI-determined short diameter of the largest ALN | 0.13831200  0.07403100 | 0.03011584 | 0.738  (0.649-0.827) | 0.649  (0.429-0.870) |
|  | 4 | CA 15-3  Lymphovascular invasion  Pathologic type of breast cancer  MRI-determined short diameter of the largest ALN | 0.59183702  0.31351220  0.00450328  0.08973054 | 0.06074684 | 0.758  (0.661-0.850) | 0.793  (0.638-0.950) |
|  | 5 | CA 15-3  MRI-determined short diameter of the largest ALN | 0.26645237  0.04717238 | 0.08722399  - | 0.749  (0.660-0.839) | 0.687  (0.490-0.883) |
| **MRI-Clinical-Radiomics Nomogram** | 1 | CA 15-3  MRI-determined short diameter of the largest ALN  DWI_ Original GLDM Small Dependence High Gray Level Emphasis  ADC_ Wavelet HHH NGTDM Contrast | 0.00241578  0.06460718  -0.44089266  0.08594007 |  | 0.876  (0.808-0.945) | 0.852  (0.689-1.000) |
|  | 2 | CA 15-3  Lymphovascular invasion  Pathologic type of breast cancer  MRI-determined short diameter of the largest ALN  MRI BI_RADS  DWI_ Original GLDM Small Dependence High Gray Level Emphasis | 0.40783246  0.39530084  0.43770842  0.10373059  -0.11705715  0.08893196 | - | 0.840  (0.762-0.919) | 0.837  (0.689-0.986) |
|  | 3 | CA 15-3  MRI-determined short diameter of the largest ALN  DWI_ Original GLDM Small Dependence High Gray Level Emphasis | 0.13831200  0.07403100  -0.17671750 | - | 0.875  (0.807-0.943) | 0.788  (0.592-0.984) |
|  | 4 | CA 15-3  Lymphovascular invasion  Pathologic type of breast cancer  MRI-determined short diameter of the largest ALN  DWI_ Original GLDM Small Dependence High Gray Level Emphasis | 0.59183702  0.31351220  0.00450328  0.08973054  -0.08747149 | - | 0.779  (0.689-0.868) | 0.778  (0.618-0.938) |
|  | 5 | CA 15-3  MRI-determined short diameter of the largest ALN  DWI_ Original GLDM Small Dependence High Gray Level Emphasis | 0.26645237  0.04717238  -0.09391285 | - | 0.781  (0.696-0.868) | 0.851  (0.695-1.000) |

DWI, diffusion-weighted imaging; GLDM, Gray Level Dependence Matrix; ADC, apparent diffusion coefficient; NGTDM, Neighbouring Gray Tone Difference Matrix; CA 15-3, carbohydrate antigen 15-3; MRI, magnetic resonance imaging; ALN, axillary lymph node; BI_RADS, breast imaging reporting and data system.
